# Deaths with preceding hospitalisations within 180 days in eight countries in sub-Saharan Africa and South Asia: A secondary descriptive analysis of the Child Health and Mortality Prevention Surveillance (CHAMPS) network

**DOI:** 10.1136/bmjopen-2025-106095

**Published:** 2026-03-23

**Authors:** Rosauro Varo, Kaitlin Cole, Zachary J Madewell, Jaime Fanjul Iglesias, Kitiezo Aggrey Igunza, Victor Akelo, Christopher Mugah, Dickens Onyango, Joyce A Were, Shabir A Madhi, Ziyaad Dangor, Siobhan Johnstone, Sanjay G Lala, Tanya Ruder, Inacio Mandomando, Milton Kincardett, Elisio G Xerinda, J Anthony G Scott, Nega Assefa, Lola Madrid, Faisel A Hassen, Yunus Edris, Ikechukwu Ogbuanu, Ima-Abasi Bassey, Solomon Samura, Abdul Salaam Sillah, Erick Kaluma, Shams El Arifeen, Rajib Biswas, Emily S Gurley, Afruna Rahman, Mohammad Zahid Hossain, Saad B Omer, Abdul Momin Kazi, Sameer M Belgaumi, Raheel Allana, Adama M Keita, Quique Bassat, Portia C Mutevedzi, Cynthia G Whitney, Chris A Rees, On behalf of the Child Health and Mortality Prevention Surveillance (CHAMPS) Network, Fatima Solomon

**Affiliations:** 1 ISGlobal, Barcelona, Spain; 2 Centro de Investigação em Saúde de Manhiça, Manhica, Mozambique; 3 Consorcio de Investigación Biomédica en Red de Epidemiología y Salud Pública (CIBERESP), Madrid, Spain; 4 Institut de Recerca Sant Joan de Deu, Hospital Sant Joan de Deu, Barcelona, Spain; 5 Emory University School of Medicine, Atlanta, Georgia, USA; 6 Global Health Center, US Centers for Disease Control and Prevention, Atlanta, Georgia, USA; 7 Center for Global Health Research, Kenya Medical Research Institute, Kisumu, Kenya; 8 Liverpool School of Tropical Medicine, Liverpool, UK; 9 Department of Epidemiology, Kisumu County Department of Health, Kisumu, Kenya; 10 South African Medical Research Council Vaccines and Infectious Diseases Analytics Research Unit, Faculty of Health Sciences, University of the Witwatersrand, Johannesburg, South Africa; 11 Wits Infectious Diseases and Oncology Research Institute, Faculty of Health Sciences, University of the Witwatersrand, Johannesburg, South Africa; 12 Department of Paediatrics & Child Health, Faculty of Health Sciences, University of the Witwatersrand, Johannesburg, South Africa; 13 Department of Infectious Disease Epidemiology and International Health, London School of Hygiene and Tropical Medicine, Keppel Street, London, UK; 14 College of Health and Medical Sciences, Haramaya University, Harar, Ethiopia; 15 Hubert Department of Global Health, Rollins School of Public Health, Emory University, Atlanta, Georgia, USA; 16 CHAMPS Sierra Leone, The Africa Research Collaborative, Freetown, Sierra Leone; 17 World Hope International, Freetown, Sierra Leone; 18 Emory University Rollins School of Public Health, Atlanta, Georgia, USA; 19 International Centre for Diarrhoeal Disease Research Bangladesh (ICDDRB), Dhaka, Bangladesh; 20 Department of Epidemiology, Johns Hopkins Bloomberg School of Public Health, Baltimore, Maryland, USA; 21 Peter O’Donnell Jr. School of Public Health, University of Texas Southwestern Medical Center, Dallas, Texas, USA; 22 Department of Pediatrics and Child Health, Aga Khan University Hospital, Karachi City, Pakistan; 23 Centre pour le Développement des Vaccins, Ministère de la Santé, Bamako, Mali; 24 ICREA, Pg. Lluís Companys 23, 08010, Barcelona, Spain; 25 Institut Clínic de Medicina I Dermatologia, Hospital Clínic de Barcelona, Barcelona, Spain; 26 Facultat de Medicina i Ciències de la Salut, Universitat de Barcelona (UB), Barcelona, Spain; 27 Task Force for Global Health, Atlanta, Georgia, USA; 28 Division of Pediatric Emergency Medicine, Emory University School of Medicine, Atlanta, Georgia, USA

**Keywords:** Child, TROPICAL MEDICINE, Neonatal intensive & critical care, Mortality

## Abstract

**Abstract:**

**Objectives:**

To describe (1) the proportion of deaths that were in recently hospitalised children and (2) causes of mortality among deceased children aged 0–59 months with preceding hospitalisations who enrolled in a mortality surveillance programme.

**Design:**

Descriptive study using prospectively collected data.

**Setting:**

Eight Child Health and Mortality Prevention Surveillance (CHAMPS) community and healthcare sites in sub-Saharan Africa and South Asia.

**Participants:**

Deaths among children aged 0–59 months enrolled in CHAMPS 2016–2023.

**Interventions:**

None.

**Primary and secondary outcome measures:**

Deaths with antecedent hospitalisations within 180 days of death. Causes of death determined by expert panels who reviewed clinical data and histopathologic and microbiologic results from postmortem minimally invasive tissue sampling.

**Results:**

CHAMPS enrolled 8548 deaths; we excluded 3688 neonates who died before discharge or ≤24 hours of birth and 482 with unclear information on antecedent hospitalisations. Out of the 4378 remaining deaths, 16.7% (95% CI 15.7% to 17.9%) were deaths that occurred within 180 days of a hospitalisation (n=733/4378). Of these, 55.7% (95% CI 52.0% to 59.3%) occurred outside healthcare facilities. Among included deaths with minimally invasive tissue sampling completed (n=337), lower respiratory tract infections (41.2%, 95% CI 36.0% to 46.7%), sepsis (39.8%, 95% CI 34.5% to 45.2%) and undernutrition (n=92, 27.3%, 95% CI 22.7% to 32.4%) were most common causes of death among cases with antecedent hospitalisations. The greatest proportion of deaths with antecedent hospital admissions occurred among cases aged 1–11 months (48.0%, 95% CI 44.4% to 51.7%), compared with those aged 0–1 months (21.7%, 95% CI 18.8% to 24.9%) and those aged 1–5 years (30.3%, 95% CI 27.0% to 33.8%). Moreover, the greatest proportion of deaths with antecedent hospital admissions occurred among infants/children with weight-for-age Z-score of <−3 (62.5%, 95% CI 56.5% to 68.0%) compared with those with weight-for-age Z-score of ≥−3 (37.5%, 95% CI 32.0% to 43.5%).

**Conclusions:**

We observed a high proportion of deaths with antecedent hospitalisations within 180 days among young children across eight sites in sub-Saharan Africa and Asia. Among those deaths, children aged 1–11 months and undernourished infants were over-represented, suggesting early follow-up as a potential point to focus targeted support and future research.

**STRENGTHS AND LIMITATIONS OF THIS STUDY**

- Using data collected prospectively in a strong epidemiological surveillance system among 4378 deaths among young children in eight regions in sub-Saharan Africa and South Asia, our study describes the most common causes of death in cases with hospitalisations within 180 days of death using a validated approach to postmortem testing (ie, minimally invasive tissue sampling).
- Based on a detailed review of each individual case by a panel of experts who assigned specific causes of death, our study includes public health recommendations to avoid future deaths among children with preceding hospitalisations.
- Our study is limited because some cases with antecedent hospitalisations may have been missed, thus leading to an underestimation of the proportion of deaths that were cases of mortality with preceding hospitalisations within 180 days of death.
- We were unable to capture reasons or durations for preceding hospitalisations to link those diagnoses to ultimate causes of mortality.

## Introduction

 There is mounting evidence that the weeks to months following hospitalisation represent a vulnerable time in the life of children, particularly in low- and middle-income countries (LMICs).^1 2^ Prior studies suggest that rates of mortality within ≤180 days of hospital discharge among children in LMICs can be as high as 3%–13% and may even exceed in-hospital case fatality rates in some settings.^1 3^

Despite growing recognition of this public health problem among young children in LMICs, healthcare workers and policymakers have largely neglected the time following hospital discharge, due partially to resource-related constraints that limit follow-up, but also insufficiency of data on the subject.^1^ Moreover, caregivers may not seek additional care following hospital discharge despite worsening illness severity for their child.^4^ Additionally, available data on childhood mortality are collected mostly from healthcare facilities, which raises concerns that community deaths are missed and not included in epidemiologic mortality estimates, despite community deaths comprising up to 30%–40% of all childhood deaths in some settings.^3 5^ Thus, targeted mortality surveillance efforts in the weeks and months following hospital discharge may present a key time to reduce deaths among children and to provide more accurate estimates of overall causes of childhood mortality.

Prior studies suggest that risk factors for mortality following hospital discharge may include unplanned discharge, or antecedent hospital diagnoses such as diarrhoea, severe anaemia, severe malnutrition, respiratory infections, HIV infection or bacteraemia.^1 6 7^ In addition, results from a recent study in Uganda, using verbal autopsies, suggest that pneumonia, sepsis, and malaria were the most common estimated causes of mortality among children following hospital discharge.^8^ There are, however, concerns regarding the accuracy of verbal autopsy in ascertaining true causes of death.^9 10^ Studies including postmortem histopathology testing of tissues of children who die following hospital discharge, in the community or after readmission, are lacking.

Here, our objectives were to describe the frequency and timing of antecedent hospitalisations within 180 days among childhood deaths, identify populations of child deaths who most commonly had antecedent hospitalisations, describe causes of mortality among children with preceding hospitalisations within 180 days of death, and describe expert-panel public health recommendations on ways that future similar deaths could be averted in the Child Health and Mortality Prevention Surveillance (CHAMPS) network.

## Methods

### Study design

We conducted a descriptive study using data that were collected as part of prospective childhood mortality surveillance in the CHAMPS network from 2016 to 2023. The study procedures of CHAMPS have been described previously.^11 12^ Briefly, CHAMPS conducts prospective mortality surveillance and extensive postmortem diagnostic testing for stillbirths and deaths among neonates, infants, and young children in catchment areas with high rates of childhood mortality in six African countries, Pakistan, and Bangladesh. The overarching objective of CHAMPS is to describe true causes of death to better inform public health interventions to reduce childhood mortality.

### Patient and public involvement

The development of the research question was informed by the burden of mortality among children with preceding hospitalisations in the selected study regions. Communities were not involved in the design or conduct of this specific study but have provided extensive input on the operations of CHAMPS in study settings.

### Study setting

CHAMPS conducts childhood mortality surveillance for deaths occurring both in healthcare facilities (ie, referral hospitals, community hospitals, and clinics) and in the community (ie, at the household level) at each site. Data for this study came from CHAMPS sites in the following regions: Baliakandi and Faridpur, Bangladesh; Kersa, Haramaya and Harar, Ethiopia; Kisumu and Siaya, Kenya; Bamako, Mali; Manhiça and Quelimane, Mozambique; Karachi, Pakistan; Makeni and Bo, Sierra Leone; and Soweto, South Africa.^13^ These sites were selected for CHAMPS because they had mortality rates among children aged <5 years of >50/1000 live births at the time that implementation activities of CHAMPS began. These sites are in countries which continue to experience a high burden of mortality among children aged <5 years, ranging from 30.6 deaths/1000 live births in Bangladesh to 94.3 deaths/1000 live births in Sierra Leone.

### Inclusion and exclusion criteria

We included deaths enrolled in CHAMPS from 2016 to 2023 that were aged 0–59 months at the time of death who underwent full postmortem examination and/or those with verbal autopsies done. We excluded stillbirths and neonates who died <24 hours of birth that enrolled in CHAMPS because mortality with preceding hospitalisation, per definition, was not possible for such cases. We also excluded neonates who were born and died in the hospital without ever being discharged and neonates who were brought to the hospital shortly after a home birth or clinic birth who died during that same hospitalisation. After extensive review of all available data, we excluded deaths that did not have adequate data to identify if there was a hospitalisation within the previous 180 days. Analysis of causes of death was limited to decedents who had minimally invasive tissue sampling (MITS) performed and the causes of death determined by a specialised panel, known as the Determination of Cause of Death (DeCoDe) panel, as described previously.^14^

When identifying preceding hospitalisations, we did not classify hospitalisations for delivery as a prior hospitalisation if the child did not receive any clinical care beyond routine newborn care. We did, however, include any hospitalisations for delivery where newborns required additional interventions as well as any hospitalisations for illness episodes. However, we were unable to decipher specific reasons for delivery-related illnesses that resulted in hospitalisation.

### Data sources

CHAMPS staff conduct active surveillance in healthcare facilities and the community using the Health and Demographic Surveillance System and community health volunteers.^15 16^ After a death among a child aged 0–59 months is identified, CHAMPS teams approach caregivers for consent to participate. Enrolled cases then have extensive demographic, clinical information, anthropometry, and verbal autopsy data collected. The verbal autopsy is conducted using the WHO Verbal Autopsy form.^17^

Additionally, for deaths identified within 24 hours (or 72 hours for bodies that are refrigerated within 24 hours of death), CHAMPS staff also ask families for consent for the MITS procedure,^12^,^1518^ which involves postmortem tissue sampling from brain, lungs, and liver using biopsy needles. Blood, cerebrospinal fluid, and oropharyngeal, nasopharyngeal, and rectal swabs are also collected. Samples undergo extensive screening for pathogens using traditional microbiological cultures (blood and cerebrospinal fluid) and more advanced molecular approaches (TaqMan array card PCR, targeting >125 pathogens). Additionally, tissues are thoroughly evaluated through histopathology and targeted immunohistochemistry testing.^19^ Specific samples undergo HIV testing by PCR, tuberculosis testing using GeneXpert, and malaria screening using rapid diagnostic tests and microscopy. Pathologists at each site and at the US Centers for Disease Control and Prevention review the samples to identify histopathologic causes of death. MITS has been shown to correlate strongly with complete diagnostic autopsies.^20^,^22^

DeCoDe panel members review all data collected in CHAMPS to determine causes of death and conditions contributing to death.^14^ The DeCoDe panel is comprised of local clinical and public health experts who review all available clinical, verbal autopsy, and postmortem data to apply a systematic approach to determine causes of death for each enrolled case. DeCoDe uses the WHO death certificate and the International Classification of Diseases, 10th Revision*,* for reporting causes of death,^14^ including assigning the underlying cause, immediate cause, and comorbid conditions contributing to death. In addition to assigning causes of death, based on what was learnt about each case, DeCoDe panels provide expert-level, consensus opinions on public health interventions that could be employed to avoid future similar deaths. This is done by selecting from a list of personal and health systems approaches that could be implemented to avert future childhood deaths. These recommendations do not imply absolute preventability, but rather are meant to inform future efforts to reduce future childhood deaths based on expert opinion. This list of health systems improvements was developed iteratively by DeCoDe panels at each site when CHAMPS began in 2016.

### Variables

For this study, we analysed discrete CHAMPS variables (eg, age at the time of death, sex, site of death, place of death, immediate cause of death, underlying cause of death, breastfeeding status, weight-for-age Z score, whether the case underwent full postmortem testing, and public health recommendations to potentially avoid future deaths based on lessons learnt from each case). Preceding hospitalisations are captured in CHAMPS as both discrete variables and in narrative fields summarising both clinical data and verbal autopsies. These summaries were reviewed by two different clinicians to identify preceding hospitalisations and to capture the age of the child at the time of that hospitalisation. Disagreements between the two reviewers were discussed until consensus was achieved. Consistent with the timeframe used in prior studies,^8 23 24^ we assessed antecedent hospitalisations ≤180 days prior to the date of death, regardless of the ultimate location of their death. As discharge against medical advice has been linked to mortality after hospital discharge in prior studies,^25^,^28^ we reviewed narratives to identify evidence of enrolled cases having been discharged against medical advice. We also attempted to capture reasons for antecedent hospitalisations and duration of those hospitalisations. However, we were unable to reliably extract this information despite review of all available clinical information for each case.

### Statistical analyses

We calculated descriptive statistics for case demographics and to determine the frequency of antecedent hospitalisations within 180 days among cases of childhood mortality. We limited our analyses on cause of death and expert-level public health recommendations to decedents who underwent MITS and DeCoDe procedures. Cases that lacked MITS data were included in the analyses on frequency and timing of mortality following preceding hospitalisation. Time to event analysis was conducted using Kaplan-Meier curves demonstrating time from hospital discharge to death and Weibull Accelerated Failure Time (AFT) models to estimate time-to-event distributions for each age group at the time of hospital admission (ie, neonates aged 0–27 days at death, infants aged 1–11 months at death, and young children aged 12–59 months at death). Parametric functions were fitted to model time to death, with shape and scale parameters estimated for each stratum (eg, by age group at the time of hospital admission). CIs were calculated using the delta method. All analyses were conducted in R using the survreg function of the survival package. Our analyses on time from discharge to death among decedents with a preceding hospitalisation were conducted using age group at the time of the antecedent hospital admission to better inform surveillance efforts by age group at an identifiable time point (ie, hospital admission). All other analyses that included age were based on the age of the child at the time of death.

We compared most common causes of death anywhere in the causal chain of death between deaths with preceding hospitalisations within 180 days and deaths without a previous hospitalisation within 180 days. We used the χ^2^ test to identify subsets of child deaths (ie, age group at the time of death, sex, site of death, place of death, breastfeeding status, weight-for-age Z score, and whether the case underwent full postmortem testing) with higher proportions of preceding hospitalisations. We conducted additional analyses using the timeframe between hospitalisation and death within 90 days as a sensitivity analysis given that some prior studies have also used that timeframe.^25^ We compared public health recommendations to potentially avert future deaths from DeCoDe panels based on review of cases that died and had preceding hospitalisations within 180 days and those that did not. All analyses were conducted using the statistical software R (V.4.3.1; R Foundation for Statistical Computing).

## Results

### Characteristics of study population

**Figure 1:**
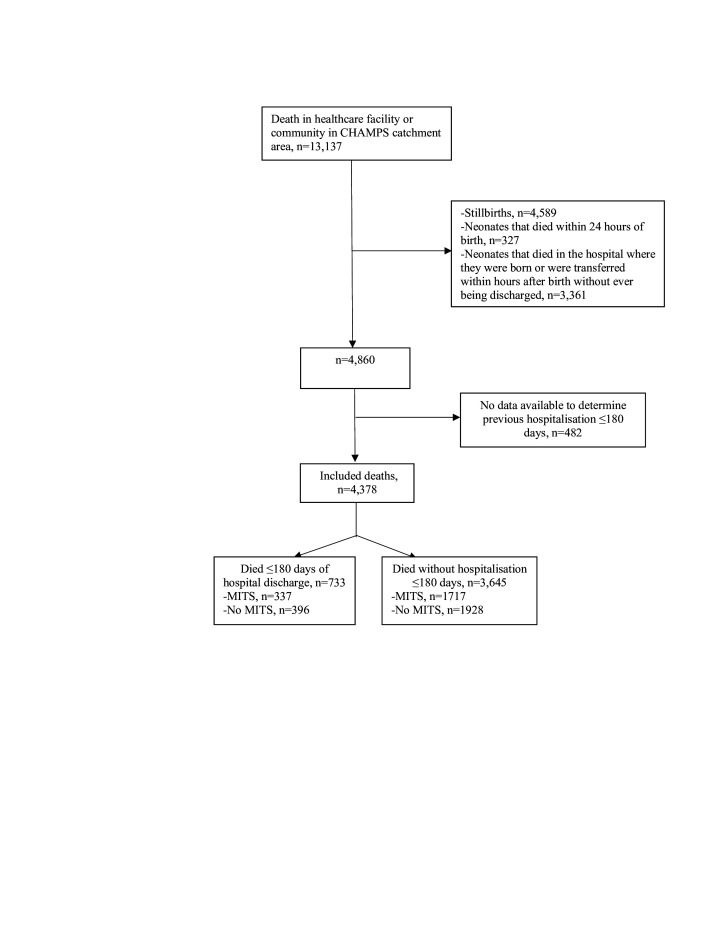
Flow diagram of included cases of mortality with preceding hospitalisations at eight sites in the Child Health and Mortality Prevention Surveillance (CHAMPS) network. MITS, minimally invasive tissue sampling.

CHAMPS identified 13 137 deaths of children aged 0–59 months within the catchment areas. After excluding stillbirths (n=4589), neonates that died within 24 hours of birth (n=327) or without ever being discharged (n=3361), and deaths without sufficient data to determine if a hospitalisation occurred in the preceding 180 days (n=482), 4378 total deaths were included in the analysis (figure 1). Out of the 4378 total deaths, 16.7% (95% CI 15.7% to 17.9%) were deaths that had a preceding hospitalisation within 180 days of death (n=733/4378). Postmortem MITS was performed for 46% (n=337/733) of cases with a preceding hospitalisation within 180 days. 62% (n=454/733) of deaths with a preceding hospitalisation occurred within 90 days of a prior hospitalisation (online supplemental table S1). The proportion of deaths with preceding hospitalisations varied by site (table 1).

**Table 1:** Description of deaths included in the analyses of mortality with preceding hospitalisations in the Child Health and Mortality Prevention Surveillance (CHAMPS) network overall and for those who had or did not have a hospitalisation within 180 days of death

	All deaths, n (%)	Had preceding hospitalisation ≤180 days before death, n (%) N=733	No preceding hospitalisation ≤180 days before death, n (%) N=3645	P value*
Age group at the time of index hospitalisation				<0.001
Neonates (aged 0–27 days)	951 (21.7)	159 (21.7)	792 (21.7)	
Infants (aged 28–364 days)	1737 (39.7)	352 (48.0)	1385 (38.0)	
Young children (aged 12–59 months)	1690 (38.6)	222 (30.3)	1468 (40.3)	
Sex				0.183
Male	2367 (54.1)	416 (56.8)	1951 (53.5)	
Female	2005 (45.8)	315 (43.0)	1690 (46.4)	
Indeterminate or ambiguous	1 (0.0)	0 (0.0)	1 (0.0)	
Unknown	5 (0.1)	2 (0.3)	3 (0.1)	
Site				<0.001
Bangladesh	459 (10.5)	131 (17.9)	328 (9.0)	
Ethiopia	864 (19.7)	96 (13.1)	768 (21.1)	
Kenya	602 (13.8)	105 (14.3)	497 (13.6)	
Mali	572 (13.1)	72 (9.8)	500 (13.7)	
Mozambique	701 (16.0)	124 (16.9)	577 (15.8)	
Pakistan	126 (2.9)	32 (4.4)	94 (2.6)	
Sierra Leone	695 (15.9)	89 (12.1)	606 (16.6)	
South Africa	359 (8.2)	84 (11.5)	275 (7.5)	
Death occurred in healthcare facility	2110 (48.2)	325 (44.3)	1785 (49.0)	0.029
Death occurred <24 hours of admission	633 (42.7)	73 (30.9)	560 (44.9)	
Death occurred 24 to <48 hours of admission	188 (12.7)	22 (9.3)	166 (13.3)	
Death occurred 48 to <72 hours of admission	106 (7.1)	23 (9.7)	83 (6.7)	
Death occurred ≥72 hours of admission	557 (37.5)	118 (50.0)	439 (35.2)	
Death occurred outside healthcare facilities	2268 (51.8)	408 (55.7)	1860 (51.0)	
Breastfeeding status	282 (6.4)	60 (8.2)	222 (6.1)	0.043
Weight-for-age Z score category†				<0.001
> −2	688 (40.7)	74 (26.0)	614 (43.7)	
−2 to −3	275 (16.3)	33 (11.5)	242 (17.2)	
<-3	726 (43.0)	178 (62.5)	548 (39.0)	
MITS status				0.604
Underwent minimally invasive tissue sampling (MITS)	2054 (46.9)	337 (46.0)	1717 (47.1)	
Verbal autopsy only (no MITS)	2324 (53.1)	396 (54.0)	1928 (52.9)	

* Calculated with χ2 testing comparing deaths with preceding hospitalisations to those without.

† Excludes neonates. Weight-for-age Z score was missing for 1739 (50.7%) infant/child cases, which is because of the inclusion of community deaths that did not enrol in full CHAMPS procedures, which includes anthropometry.

Among the 733 deaths with preceding hospitalisations within 180 days, 159 (21.7%, 95% CI 18.8% to 24.9%) were neonates aged 0–27 days at the index hospitalisation, 352 (48.0%, 95% CI 44.4% to 51.7%) were infants aged 28–365 days at the index hospitalisation, and 222 (30.3%, 95% CI 27.0% to 33.8%) were children aged 12–59 months at the index hospitalisation (table 1). Moreover, the greatest proportion of deaths with antecedent hospital admissions occurred among infants/children with weight-for-age Z-score of <−3 (62.5%, 95% CI 56.5% to 68.0%) compared with those with weight-for-age Z-score of ≥−3 (37.5%, 95% CI 32.0% to 43.5%).

Over half of deaths among children with preceding hospitalisations within 180 days occurred outside of a healthcare facility (55.7%, 95% CI 52.0% to 59.3%, n=408/733; table 1), a higher proportion than among deaths without a previous hospitalisation (51.0%, 95% CI 49.4% to 52.7%, n=1860/3,645; p=0.024). 94% (n=689/733) of mortality cases with preceding hospitalisation within 180 days had only one (range 1–3) identifiable prior hospitalisation within 180 days. Discharge against medical advice in the preceding hospitalisation occurred in 7.2% (95% CI 5.5% to 9.4%, n=53/733) of these deaths.

### Timing of deaths after hospital discharge for cases of mortality with prior hospitalisations

**Figure 2:**
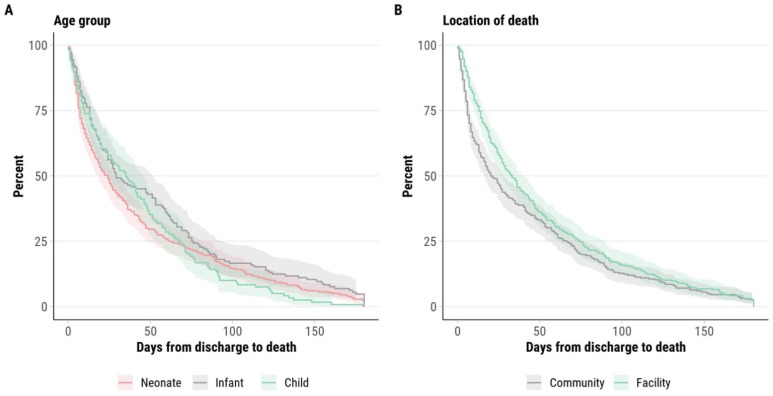
Kaplan-Meier curve demonstrating time from discharge to death stratified by age group at time of hospitalisation and location of death among children who died in the Child Health and Mortality Prevention Surveillance network.

Among mortality cases with preceding hospitalisations within 180 days, 74.2% (n=544/733) had available specific dates for both the preceding hospital discharge and death. Among these, 52.4% (n=285/544) of deaths occurred 1–30 days after discharge, 18.2% (n=99/544) occurred 31–60 days, 11.9% (n=65/544) occurred 61–90 days, and 17.5% (n=95/544) occurred 91–180 days following discharge. Time from hospital discharge to death varied by age category at the time of hospitalisation (figure 2). Among those aged 0–27 days (neonates) during their index hospitalisation, the median time from discharge to death was 24 days (IQR 7, 62 days), compared with 29 days (IQR 13, 75 days) for infants, and 35 days (IQR 10, 68 days) for children, although the difference was not statistically significant (p=0.057). Among mortality cases with preceding hospitalisations within 180 days, the AFT model estimated the median time from discharge to death as 24 days for deaths that occurred in the community and 35 days for deaths that occurred in healthcare facilities (online supplemental figure S1).

### Comparison of causes of death for mortality cases with preceding hospitalisations within 180 days to those without preceding hospitalisations within 180 days of death

**Figure 3:**
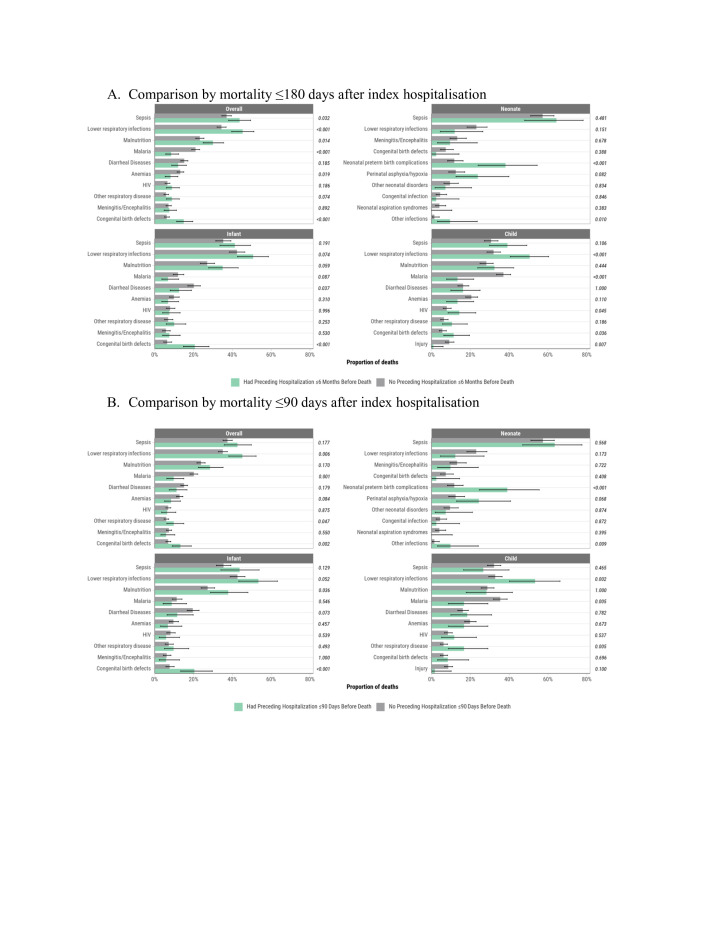
Comparison of the proportion of deaths from specific causes among young children 0–59 months of age at the time of death with preceding hospitalisation before death to those without preceding hospitalisations overall and by age group at the time of death (N=2054).

DeCoDe panel-attributed causes of death varied between cases of mortality with prior hospitalisations within 180 days and cases without preceding hospitalisations within 180 days. Examining all age groups combined, lower respiratory infections (p<0.001), sepsis (p=0.032), malnutrition (p=0.014) and birth defects (p<0.001) were more common in the causal chain of death for cases of mortality with preceding hospitalisations compared with deaths without preceding hospitalisations (figure 3A). Conversely, malaria (p<0.001) and anaemia (p=0.02) were more common in the causal chain of death in cases without preceding hospitalisations compared with mortality cases that had preceding hospitalisations within 180 days. Among neonates, neonatal preterm birth complications (p<0.001) and other infections (p=0.010) were more commonly in the causal chain of death for cases of mortality that had preceding hospitalisations within 180 days than those without preceding hospitalisations. Among infant deaths, birth defects were more commonly in the causal chain of death among cases of mortality with preceding hospitalisations than those without preceding hospitalisations (p<0.001). Among child deaths, lower respiratory tract infections (p<0.001), HIV (p=0.045), and birth defects (p=0.036) were more common among cases of mortality with preceding hospitalisations than among deaths without preceding hospitalisations. Similar observations were evident among cases of mortality with preceding hospitalisations that occurred within ≤90 days of hospitalisation (figure 3B).

Causes of mortality among cases that had preceding hospitalisations within 180 days also varied by country, with sepsis-related deaths being most common in Bangladesh and lower respiratory tract infections being most common in South Africa (online supplemental figure S5). Furthermore, when restricting analyses of mortality among cases with preceding hospitalisations with HIV in the causal chain to the four CHAMPS sites where HIV is more common (ie, Kenya, South Africa, Sierra Leone, and Mozambique), HIV was present in 14.3% (n=15/105) cases of mortality with preceding hospitalisations within 180 days and 7.4% (n=52/702) of cases without prior hospitalisations among deaths for children aged 12–59 months (p=0.03).

The immediate cause of death was more likely to be sepsis or lower respiratory infections in cases of mortality with preceding hospitalisations than among cases without preceding hospitalisation ≤180 days (online supplemental figure S2). The underlying cause of death was more likely to be birth defects in mortality cases among infants at the time of death who had preceding hospitalisations (online supplemental figure S3). Lower respiratory infections and malnutrition were more commonly identified as comorbid conditions in cases of mortality with preceding hospitalisations (online supplemental figure S4).

### Expert-panel public health recommendations to avert future deaths based on review of mortality among children with preceding hospitalisations

Most deaths with preceding hospitalisations within 180 days and within 90 days were deemed possibly preventable through expert opinion by the DeCoDe panels (table 2 and online supplemental table S2). Compared with deaths without preceding hospitalisations, cases of mortality with prior hospitalisations within 180 days were more likely to lead to expert panel public health recommendations for improved clinical care to avert similar deaths in the future (p=0.003). DeCoDe panels more commonly recommended nutritional support as a public health intervention to avert future deaths based on review of mortality cases with preceding hospitalisations compared with cases without preceding hospitalisations (p<0.001). DeCoDe panels more commonly recommended improved health education based on review of cases without a preceding hospitalisation compared with cases of mortality with prior hospitalisations (p=0.025). In stratified analyses, public health recommendations to potentially avert future deaths varied by age group at the time of death (online supplemental table S3).

**Table 2:** Comparison of expert-panel opinion on potential preventability and public health recommendations to potentially avoid future deaths based on lessons learnt from each case between children aged 0–59 months who had died and had preceding hospitalisation within 180 days compared with cases that died but did not have prior hospitalisation within 180 days

	All cases		No preceding hospitalisation ≤180 days before death		Had preceding hospitalisation ≤180 days before death		P value*
	n	% (95% CI)	n	% (95% CI)	n	% (95% CI)	
Expert panel opinion deemed death to be preventable†							0.016
Yes	1599	86.3 (84.6 to 87.8)	1347	87.2 (85.4 to 88.8)	252	81.8 (77.0 to 85.9)	
No	254	13.7 (12.2 to 15.4)	198	12.8 (11.2 to 14.6)	56	18.2 (14.1 to 23.0)	
Recommendations to potentially avert future similar deaths†							
Improved antenatal/obstetric care	133	9.2 (7.8 to 10.8)	109	8.9 (7.4 to 10.7)	24	10.8 (7.2 to 15.8)	0.437
Clinical management	868	60.0 (57.4 to 62.6)	714	58.3 (55.5 to 61.1)	154	69.4 (62.8 to 75.3)	0.003
Health-seeking behaviour	667	46.1 (43.5 to 48.7)	576	47.1 (44.2 to 49.9)	91	41.0 (34.5 to 47.8)	0.111
HIV prevention	95	6.6 (5.4 to 8.0)	80	6.5 (5.2 to 8.1)	15	6.8 (4.0 to 11.1)	1.000
Health education	610	42.2 (39.6 to 44.8)	532	43.5 (40.7 to 46.3)	78	35.1 (28.9 to 41.8)	0.025
Nutritional support	349	24.1 (22.0 to 26.4)	271	22.1 (19.9 to 24.6)	78	35.1 (28.9 to 41.8)	<0.001
Infection prevention	361	25.0 (22.8 to 27.3)	306	25.0 (22.6 to 27.5)	55	24.8 (19.4 to 31.1)	1.000
Vaccinations	87	6.0 (4.9 to 7.4)	67	5.5 (4.3 to 6.9)	20	9.0 (5.7 to 13.8)	0.060
Family planning	38	2.6 (1.9 to 3.6)	32	2.6 (1.8 to 3.7)	6	2.7 (1.1 to 6.1)	1.000
Transport system	50	3.5 (2.6 to 4.6)	40	3.3 (2.4 to 4.5)	10	4.5 (2.3 to 8.4)	0.467

* Comparison between cases with preceding hospitalisation ≤180 days to those with no preceding hospitalisation ≤180 days of death.

† Public health recommendations to potentially avoid future deaths based on lessons learnt from each case were available for 1853 (42.3%) of the 4378 deaths, including 1545 deaths with no preceding hospitalisation ≤180 days before death, 197 deaths that had preceding hospitalisation ≤90 days before death and 308 deaths that had preceding hospitalisation ≤180 days before death.

## Discussion

In our study of >4000 deaths among children aged 0–59 months, one in six had been hospitalised within the preceding 180 days. Most cases of mortality had preceding hospitalisations within 2 months of the time of death. Over half of mortality occurred outside medical facilities among cases with preceding hospitalisations within 180 days. Common causes of mortality in cases with preceding hospitalisations included lower respiratory tract infections, sepsis, birth defects, and malnutrition. Expert panels more commonly recommended improved clinical management as a public health intervention to avert future deaths based on review of mortality cases with preceding hospitalisations within 180 days compared with cases without a preceding hospitalisation.

We found that the median time to death following hospital discharge was 35 days or less in all age groups at the time of the previous hospital admission, indicating that for many deaths the time immediately after hospitalisation is a critical window for potential interventions to avoid deaths. Our finding aligns with prior studies in Kenya, Uganda, and Mozambique that suggest the median time from hospital discharge to death ranged from 28 days to 1.7 months.^25 29 30^ Enhanced surveillance of young children following hospital discharge to identify children whose clinical status may deteriorate may be most effective during the first 60 days after discharge, a period our data and others suggest is a vulnerable time. Moreover, as over half of the cases of mortality that had preceding hospitalisations occurred outside of healthcare facilities, preventing future deaths may require community follow-up and targeted interventions that might begin at the time of hospital discharge.

Consistent with prior studies that have used verbal autopsy to determine causes of death, deaths with preceding hospitalisations evaluated in our analysis using postmortem histopathologic testing found that acute causes such as lower respiratory infections and sepsis often caused death.^6^,^8^ In contrast to prior investigations, however, we used postmortem tissue sampling and comprehensive diagnostic testing to describe causes of death, which has been shown to be comparable in accuracy to complete diagnostic autopsies.^20^,^22^ Our methods, which included determination of underlying, comorbid, and immediate causes for each death, showed that mortality among cases with preceding hospitalisations was commonly due to conditions that tend to be chronic in young children such as birth defects, complications of prematurity, and undernutrition. These underlying causes of death are often associated with acute infectious causes of death like sepsis and respiratory infections. Prior studies have shown that the presence of malnutrition during the antecedent hospitalisation confers greater risk of subsequent mortality after discharge,^1 3^ but our study provides evidence of malnutrition as a cause of mortality in cases that had preceding hospitalisations.

We found that expert panels frequently recommended improved clinical management as a potential public health intervention to avert future similar deaths, which is in line with prior studies using data from the CHAMPS network that have illustrated that approximately three of every four deaths are deemed preventable through the same by expert opinion.^31^ Although our study cannot link prior hospitalisation data to causes of death, considering the identified causes of death among cases of mortality with preceding hospitalisations, it may be important to improve the continuum of clinical care during the hospitalisation but also during follow-up. Missed diagnoses and suboptimal adherence to clinical care guidelines may contribute to mortality in diverse settings in sub-Saharan Africa and South Asia.^32^,^34^ Better health-seeking was the second most common public health recommendation given by expert panels for avoiding future cases of mortality after review of cases of mortality among children with preceding hospitalisations. Thus, additional interventions to enhance healthcare seeking among caregivers of young children may be warranted.^35^ Ultimately, future prospective studies that are designed to fully assess the quality of clinical care during index hospitalisations coupled with patient follow-up are warranted to better link suboptimal clinical care to mortality following hospital discharge.

### Limitations

The results of our study must be interpreted with acknowledgement of their limitations. Our identification of cases of mortality among cases with preceding hospitalisations was limited by the data available within CHAMPS, and other antecedent hospitalisations among cases of mortality may have been missed due to insufficient information about a previous hospitalisation, thus leading to an under-estimate of the proportion of deaths that were cases of mortality that had preceding hospitalisations. We mitigated this risk through review of discrete variables filled out by CHAMPS staff who reviewed all available clinical records as well as narrative fields including verbal autopsies in which caregivers were asked about preceding healthcare encounters. We were also unable to distinguish between delivery-related hospitalisations for neonates and acute illnesses for neonates as we could not determine reasons for antecedent hospitalisations. We were also unable to ascertain reasons that children were taken home from the hospital against medical advice, although these accounted for only a small proportion of deaths. For these reasons, future studies exploring the family experience and healthcare-specific performance for each case should be performed. Moreover, we were unable to completely capture reasons or durations for preceding hospitalisations to link those diagnoses to ultimate causes of mortality. Nevertheless, our study, does have the key piece of information missing from prior studies, which is postmortem-determined causes of death among CHAMPS-enrolled decedents, including those with a preceding hospitalisation within 180 days.

Given that CHAMPS collects data only on young children who have died, this study does not allow for direct comparisons between children who died compared with those who survived. Some of our subgroup analyses by age or by CHAMPS site may lead to potential instability of significance due to the diminution of number of cases, which may affect the generalisability of our results. Thus, future single-centre studies may be indicated to better understand causes of mortality with preceding hospitalisations by CHAMPS site with larger sample sizes. Although deaths included in this study came from regions with high burden of childhood mortality in eight countries in sub-Saharan Africa and South Asia; our sample may not represent other, lower-mortality areas in CHAMPS countries or these regions. Finally, although we included deaths from several sites, our sample size of 733 deaths with preceding hospitalisations within 180 days limits generalisability of our findings.

### Conclusions

Preceding hospitalisations were common among young children who died across eight sites in sub-Saharan Africa and South Asia. Causes of death among cases with preceding hospitalisations were commonly attributable to lower respiratory infections, sepsis, malnutrition, prematurity complications, and birth defects. Preceding hospitalisations were most common among infant and young child deaths with undernutrition. Thus, it may be important to develop targeted interventions for closer follow-up to improve the continuum of clinical care after hospital discharge for these at high-risk populations, though further investigation is warranted to understand specific aspects of clinical management that may avoid future deaths. Moreover, additional studies are warranted to assess the potential association between specific reasons for hospitalisation in index hospitalisations and postmortem determined causes of death to further inform interventions to reduce potentially preventable future cases of mortality among children with preceding hospitalisations.

## Supplementary material

10.1136/bmjopen-2025-106095 online supplemental file 1

## Data Availability

Data are available on reasonable request.
