# Supplementary material for: Deaths with preceding hospitalisations within 180 days in eight countries in sub-Saharan Africa and South Asia: A secondary descriptive analysis of the Child Health and Mortality Prevention Surveillance (CHAMPS) network
Source: BMJ Open. 2026 Mar 23;16(3):e106095. doi: 10.1136/bmjopen-2025-106095 (PMC13034342; doi:10.1136/bmjopen-2025-106095)
Supplement: online supplemental file 1 [file bmjopen-16-3-s001.docx]

**Figure S1**. Weibull Accelerated Failure Time (AFT) models demonstrating time from discharge to death stratified by age group at time of hospitalization and location of death among children aged 0-59 months who died in the Child Health and Mortality Prevention Surveillance (CHAMPS) network

**
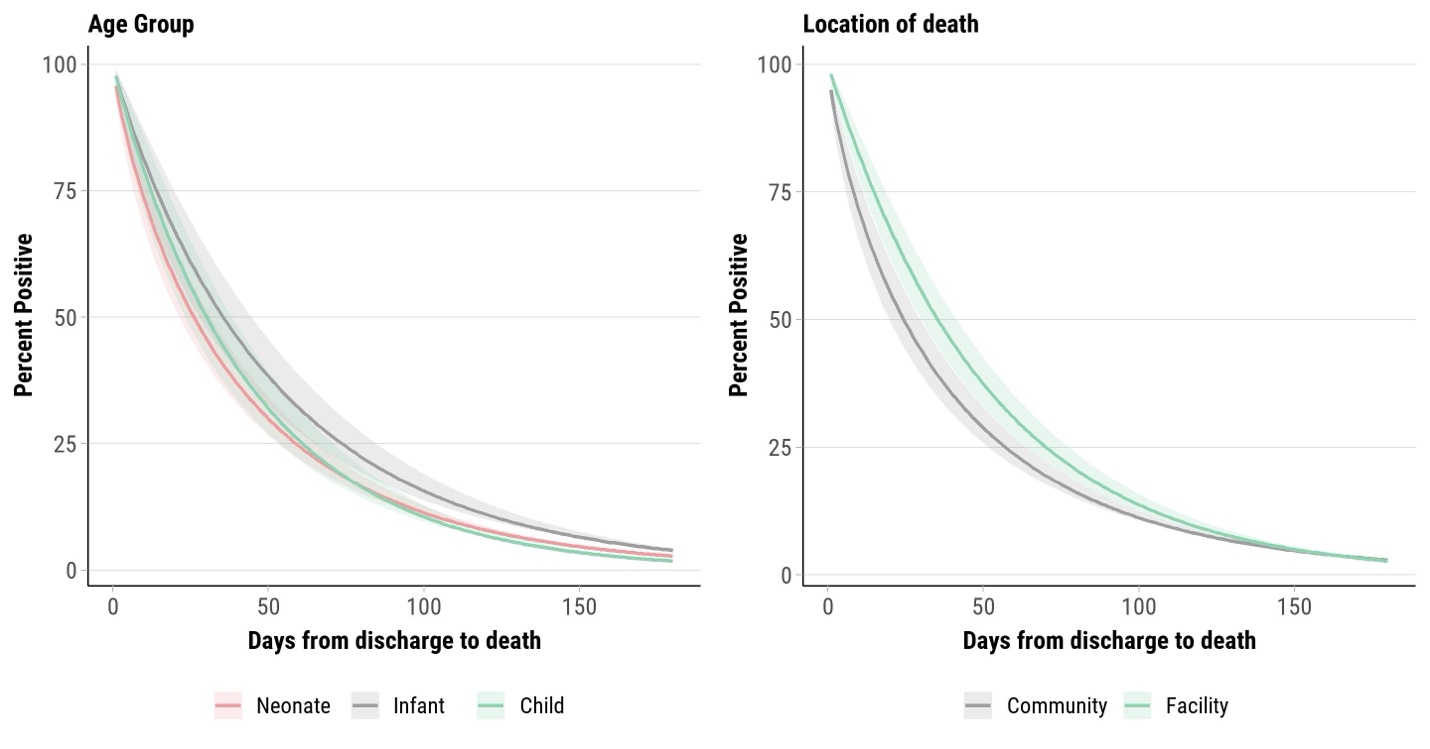
**

**Figure S2.** Comparison of immediate causes of death with preceding hospitalization ≤180 days before death to those without preceding hospitalizations overall and by age group at the time of death


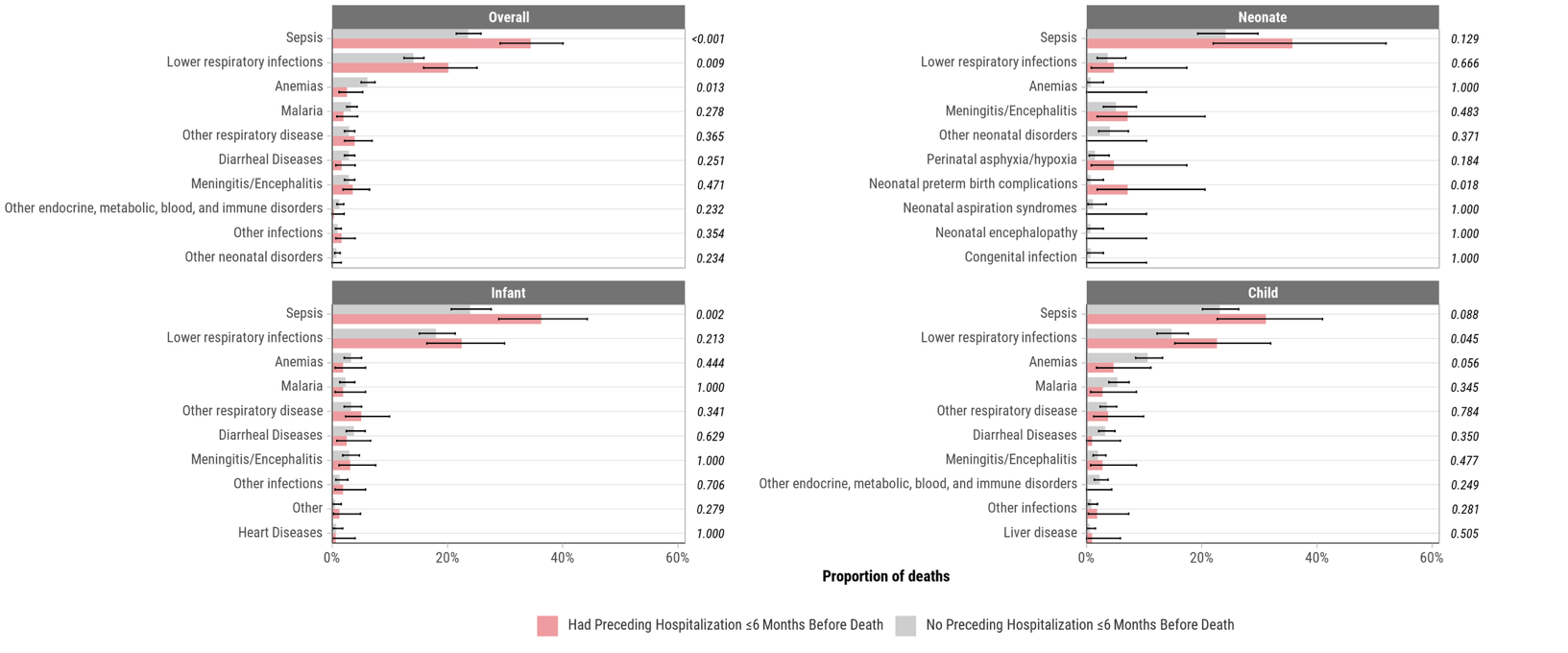


**Figure S3.** Comparison of underlying causes of death with preceding hospitalization ≤180 days before death to those without preceding hospitalizations overall and by age group at the time of death

**
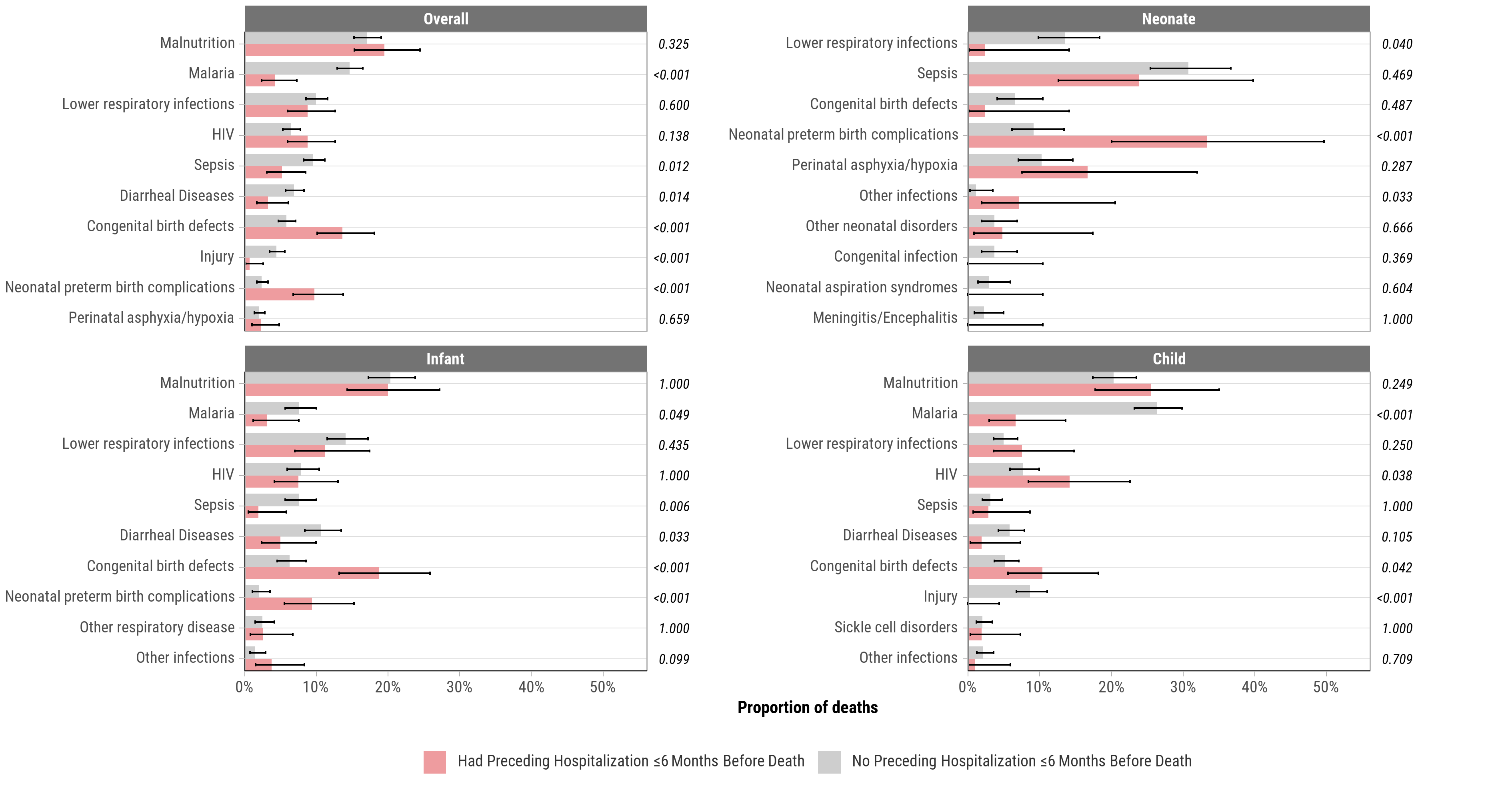
**

**Figure S4.** Comparison co-morbid conditions in deaths with preceding hospitalization ≤180 days before death to those without preceding hospitalizations overall and by age group at the time of death

**
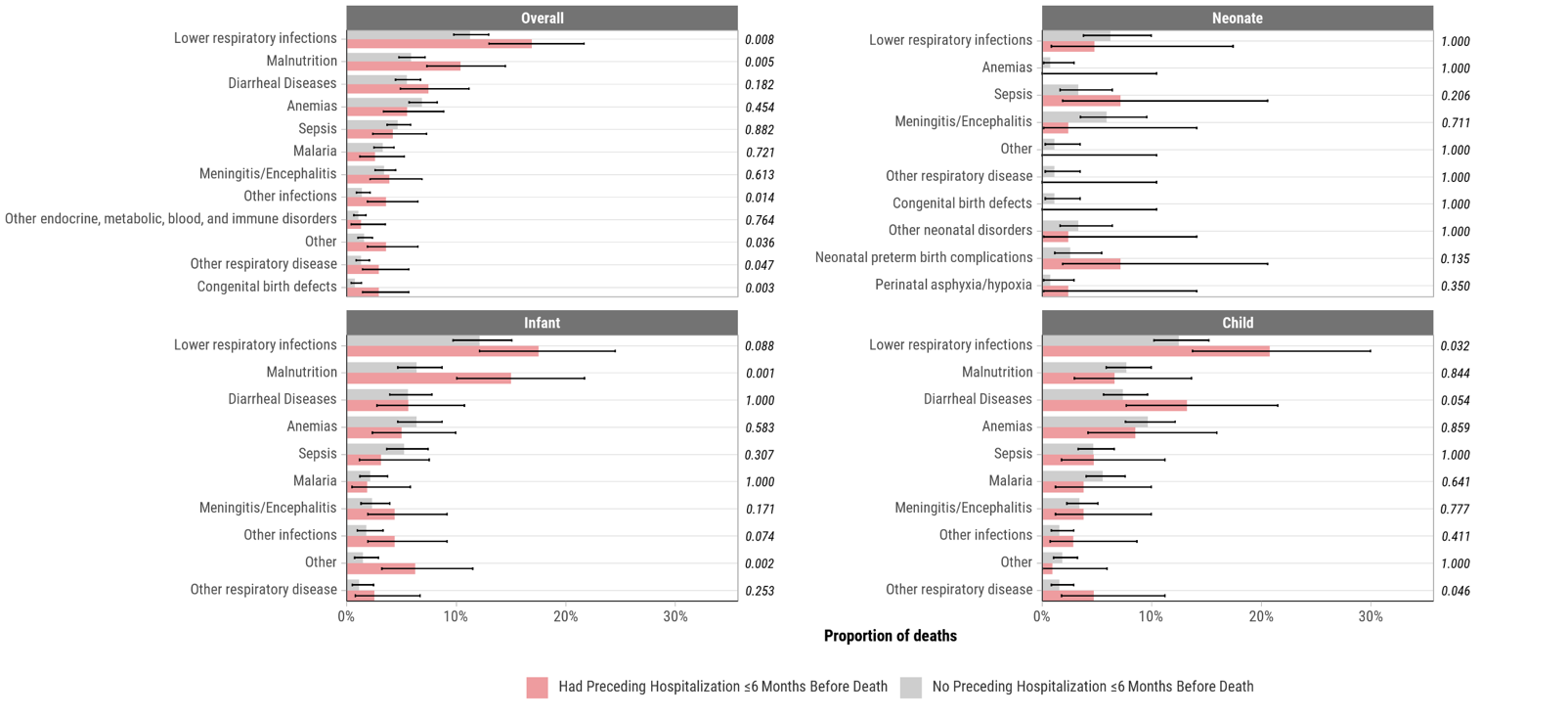
**

**Figure S5.** Comparison of causes of mortality among children with preceding hospitalizations ≤180 days by Child Health and Mortality Prevention Surveillance (CHAMPS) site.


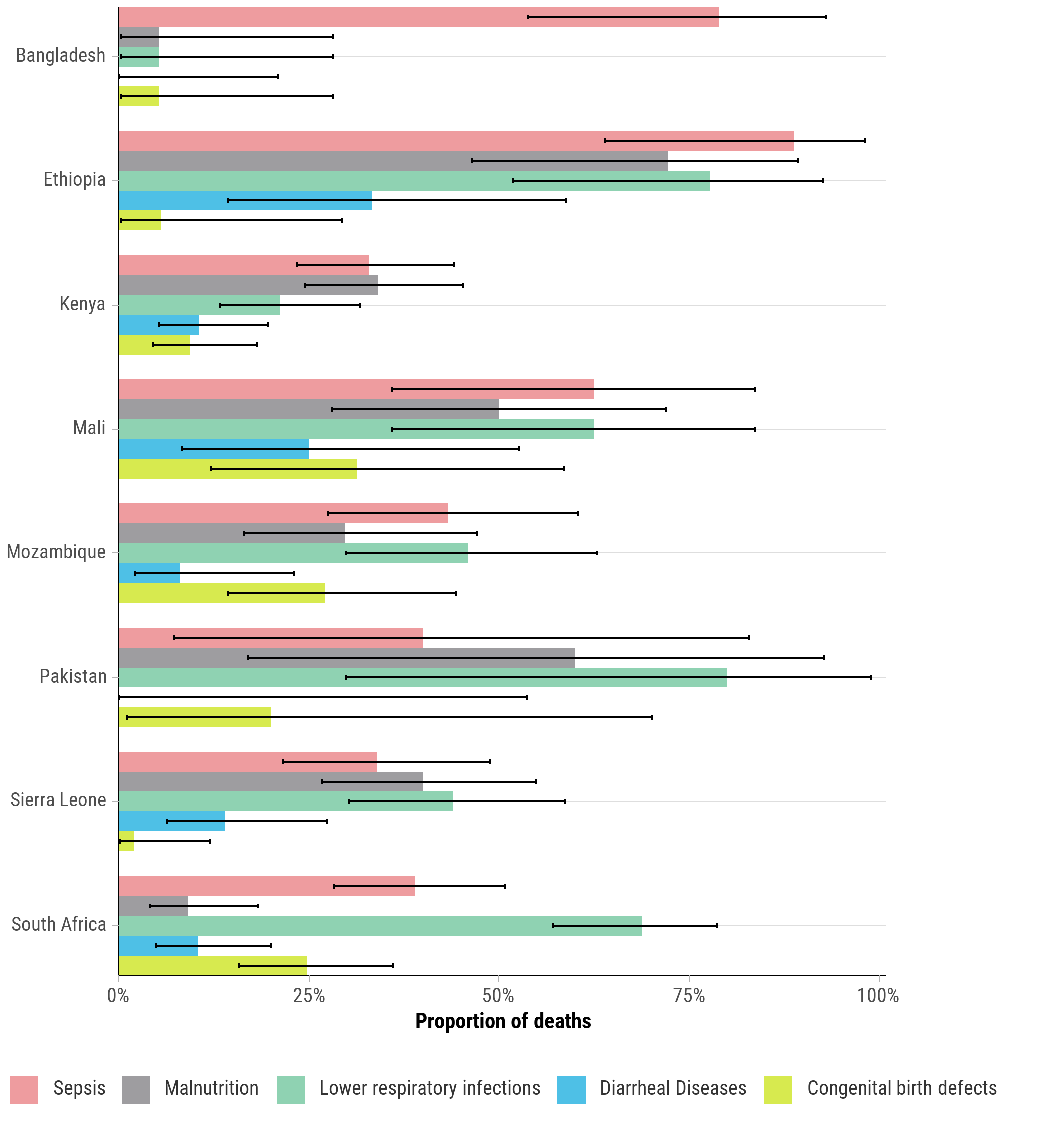


**Table S1**. Description of cases included in the analyses of mortality among children with prior hospitalizations in the Child Health and Mortality Prevention Surveillance (CHAMPS) network restricted to cases that died within 90 days of hospital discharge (N=454)

|  | **n (%)** |
| --- | --- |
| **Age Group at the Time of Death** |  |
| Neonates (aged 0-27 days) | 149 (32.8) |
| Infants (aged 28-365 days) | 195 (43.0) |
| Young children (aged 12-59 months) | 110 (24.2) |
| **Sex** |  |
| Male | 254 (55.9) |
| Female | 199 (43.8) |
| Unknown | 1 (0.2) |
| **Site** |  |
| Bangladesh | 97 (21.4) |
| Ethiopia | 29 (6.4) |
| Kenya | 63 (13.9) |
| Mali | 63 (13.9) |
| Mozambique | 76 (16.7) |
| Pakistan | 16 (3.5) |
| Sierra Leone | 67 (14.8) |
| South Africa | 55 (12.1) |
| **Death Occurred in Healthcare Facility** | 214 (47.1) |
| Death occurred <24 hours of admission | 58 (35.6) |
| Death occurred 24 to <48 hours of admission | 12 (7.4) |
| Death occurred 48 to <72 hours of admission | 17 (10.4) |
| Death occurred ≥72 hours of admission | 76 (46.6) |
| **Death Occurred Outside Healthcare Facilities** | 240 (52.9) |
| **HIV status** |  |
| Living with HIV | 17 (3.7) |
| No HIV/Unclear | 437 (96.3) |
| **Breastfeeding status** | 50 (11.0) |
| **Weight-for-Age Z Score Category** |  |
| > -2 | 138 (61.9) |
| -2 to -3 | 25 (11.2) |
| <-3 | 60 (26.9) |
| **MITS status** |  |
| Underwent minimally invasive tissue sampling (MITS) | 223 (49.1) |
| Verbal autopsy only (No MITS) | 231 (50.9) |

**Table S2.** Comparison of public health recommendations and approaches by which future similar deaths could potentially be averted between children aged 0-59 months who died and had preceding hospitalizations ≤90 days compared to cases that died but did not have preceding hospitalizations ≤90 days

|  | **All cases** | | **No Preceding Hospitalization ≤90 Days Before Death** | | **Had Preceding Hospitalization ≤90 Days Before Death** | | **P-value*** |
| --- | --- | --- | --- | --- | --- | --- | --- |
|  | **n** | **% (95% CI)** | **n** | **% (95% CI)** | **n** | **% (95% CI)** |  |
| **Deaths deemed to be preventable***** |  |  |  |  |  |  | 0.070 |
| Yes | 1599 | 86.3 (84.6, 87.8) | 1347 | 87.2 (85.4, 88.8) | 162 | 82.2 (76.0, 87.2) |  |
| No | 254 | 13.7 (12.2, 15.4) | 198 | 12.8 (11.2, 14.6) | 35 | 17.8 (12.8, 24.0) |  |
| **Recommendations****** |  |  |  |  |  |  |  |
| Improved antenatal/obstetric care | 133 | 9.2 (7.8, 10.8) | 109 | 8.9 (7.4, 10.7) | 20 | 14.1 (9.0, 21.2) | 0.064 |
| Clinical management | 868 | 60.0 (57.4, 62.6) | 714 | 58.3 (55.5, 61.1) | 107 | 75.4 (67.3, 82.0) | <0.001 |
| Health-seeking behavior | 667 | 46.1 (43.5, 48.7) | 576 | 47.1 (44.2, 49.9) | 53 | 37.3 (29.5, 45.9) | 0.035 |
| HIV prevention | 95 | 6.6 (5.4, 8.0) | 80 | 6.5 (5.2, 8.1) | 5 | 3.5 (1.3, 8.4) | 0.221 |
| Health education | 610 | 42.2 (39.6, 44.8) | 532 | 43.5 (40.7, 46.3) | 47 | 33.1 (25.6, 41.6) | 0.023 |
| Nutritional support | 349 | 24.1 (22.0, 26.4) | 271 | 22.1 (19.9, 24.6) | 41 | 28.9 (21.7, 37.2) | 0.088 |
| Infection prevention | 361 | 25.0 (22.8, 27.3) | 306 | 25.0 (22.6, 27.5) | 39 | 27.5 (20.5, 35.7) | 0.591 |
| Vaccinations | 87 | 6.0 (4.9, 7.4) | 67 | 5.5 (4.3, 6.9) | 13 | 9.2 (5.2, 15.5) | 0.114 |
| Family planning | 38 | 2.6 (1.9, 3.6) | 32 | 2.6 (1.8, 3.7) | 5 | 3.5 (1.3, 8.4) | 0.721 |
| Transport system | 50 | 3.5 (2.6, 4.6) | 40 | 3.3 (2.4, 4.5) | 7 | 4.9 (2.2, 10.3) | 0.432 |

*Comparison between cases with preceding hospitalization ≤90 days to those with no preceding hospitalization ≤90 days of death

***Preventability determinations were available for 1,853 (42.3%) of the 4378 deaths, including 1,545 deaths with no preceding hospitalization ≤180 days before death, 197 deaths that had preceding hospitalization ≤90 days before death, and 308 deaths that had preceding hospitalization ≤180 days before death.

****Recommendations for how to prevent deaths were available for 1,446 (90.4%) of the 1,599 preventable deaths, including 1224 (90.9%) of 1,347 deaths with no preceding hospitalization ≤180 days before death, 142 (87.7%) of 162 deaths that had preceding hospitalization ≤90 days before death, and 222 (88.1%) of 252 deaths that had preceding hospitalization ≤180 days before death.

**Table S3**. Comparison of public health recommendations and approaches by which future deaths potentially be averted between deaths with preceding hospitalizations ≤180 days compared to those that did not by age group at the time of death

|  | **All cases** | | **No Preceding Hospitalization ≤180 Days Before Death** | | **Had Preceding Hospitalization ≤90 Days Before Death** | | **P-value*** | **Had Preceding Hospitalization ≤180 Days Before Death** | | **P-value**** |
| --- | --- | --- | --- | --- | --- | --- | --- | --- | --- | --- |
| ***Neonatal Deaths*** | | | | | | | | | | |
|  | **n** | **% (95% CI)** | **n** | **% (95% CI)** | **n** | **% (95% CI)** |  | **n** | **% (95% CI)** |  |
| **Deaths deemed to be preventable** |  |  |  |  |  |  | 0.470 |  |  | 0.626 |
| Yes | 261 | 85.6 (81.0, 89.2) | 225 | 84.9 (79.9, 88.9) | 36 | 92.3 (78.0, 98.0) |  | 36 | 90.0 (75.4, 96.7) |  |
| No | 44 | 14.4 (10.8, 19.0) | 40 | 15.1 (11.1, 20.1) | 3 | 7.7 (2.0, 22.0) |  | 4 | 10.0 (3.3, 24.6) |  |
| **Recommendations*** |  |  |  |  |  |  |  |  |  |  |
| ANC/Obstetric Care | 79 | 33.9 (27.9, 40.4) | 68 | 34.0 (27.6, 41.1) | 11 | 33.3 (18.6, 51.9) | 1.000 | 11 | 33.3 (18.6, 51.9) | 1.000 |
| Clinical Management | 155 | 66.5 (60.0, 72.5) | 126 | 63.0 (55.9, 69.6) | 29 | 87.9 (70.9, 96.0) | 0.005 | 29 | 87.9 (70.9, 96.0) | 0.005 |
| Health-seeking Behavior | 108 | 46.4 (39.9, 53.0) | 95 | 47.5 (40.4, 54.6) | 13 | 39.4 (23.4, 57.8) | 0.453 | 13 | 39.4 (23.4, 57.8) | 0.453 |
| HIV prevention | 3 | 1.3 (0.3, 4.0) | 3 | 1.5 (0.4, 4.7) | 0 | 0.0 (0.0, 13.0) | 1.000 | 0 | 0.0 (0.0, 13.0) | 1.000 |
| Health Education | 55 | 23.6 (18.4, 29.7) | 47 | 23.5 (17.9, 30.1) | 8 | 24.2 (11.7, 42.6) | 1.000 | 8 | 24.2 (11.7, 42.6) | 1.000 |
| Nutritional Support | 21 | 9.0 (5.8, 13.6) | 17 | 8.5 (5.2, 13.5) | 4 | 12.1 (4.0, 29.1) | 0.511 | 4 | 12.1 (4.0, 29.1) | 0.511 |
| Infection prevention | 59 | 25.3 (20.0, 31.5) | 51 | 25.5 (19.7, 32.2) | 8 | 24.2 (11.7, 42.6) | 1.000 | 8 | 24.2 (11.7, 42.6) | 1.000 |
| Vaccinations | 6 | 2.6 (1.1, 5.8) | 5 | 2.5 (0.9, 6.1) | 1 | 3.0 (0.2, 17.5) | 1.000 | 1 | 3.0 (0.2, 17.5) | 1.000 |
| Family planning | 10 | 4.3 (2.2, 8.0) | 9 | 4.5 (2.2, 8.6) | 1 | 3.0 (0.2, 17.5) | 1.000 | 1 | 3.0 (0.2, 17.5) | 1.000 |
| Transport system | 12 | 5.2 (2.8, 9.0) | 10 | 5.0 (2.6, 9.3) | 2 | 6.1 (1.1, 21.6) | 0.681 | 2 | 6.1 (1.1, 21.6) | 0.681 |
| ***Infant Deaths*** | | | | | | | | | | |
| **Deaths deemed to be preventable** |  |  |  |  |  |  | 0.236 |  |  | 0.147 |
| Yes | 622 | 83.5 (80.6, 86.0) | 497 | 84.5 (81.3, 87.3) | 78 | 79.6 (70.0, 86.8) |  | 125 | 79.6 (72.3, 85.5) |  |
| No | 123 | 16.5 (14.0, 19.4) | 91 | 15.5 (12.7, 18.7) | 20 | 20.4 (13.2, 30.0) |  | 32 | 20.4 (14.5, 27.7) |  |
| **Recommendations**** |  |  |  |  |  |  |  |  |  |  |
| ANC/Obstetric Care | 36 | 6.6 (4.7, 9.1) | 27 | 6.2 (4.2, 9.0) | 6 | 9.2 (3.8, 19.7) | 0.416 | 9 | 8.3 (4.1, 15.5) | 0.396 |
| Clinical Management | 313 | 57.2 (52.9, 61.4) | 243 | 55.5 (50.7, 60.2) | 47 | 72.3 (59.6, 82.3) | 0.010 | 70 | 64.2 (54.4, 73.0) | 0.106 |
| Health-seeking Behavior | 239 | 43.7 (39.5, 48.0) | 200 | 45.7 (40.9, 50.5) | 20 | 30.8 (20.2, 43.6) | 0.031 | 39 | 35.8 (27.0, 45.6) | 0.067 |
| HIV prevention | 47 | 8.6 (6.4, 11.3) | 40 | 9.1 (6.7, 12.3) | 2 | 3.1 (0.5, 11.6) | 0.146 | 7 | 6.4 (2.8, 13.2) | 0.448 |
| Health Education | 223 | 40.8 (36.6, 45.0) | 187 | 42.7 (38.0, 47.5) | 20 | 30.8 (20.2, 43.6) | 0.079 | 36 | 33.0 (24.5, 42.8) | 0.081 |
| Nutritional Support | 152 | 27.8 (24.1, 31.8) | 109 | 24.9 (21.0, 29.3) | 23 | 35.4 (24.2, 48.3) | 0.095 | 43 | 39.4 (30.4, 49.3) | 0.004 |
| Infection prevention | 120 | 21.9 (18.6, 25.7) | 90 | 20.5 (16.9, 24.7) | 21 | 32.3 (21.5, 45.2) | 0.038 | 30 | 27.5 (19.6, 37.0) | 0.122 |
| Vaccinations | 35 | 6.4 (4.6, 8.9) | 29 | 6.6 (4.6, 9.5) | 2 | 3.1 (0.5, 11.6) | 0.407 | 6 | 5.5 (2.3, 12.1) | 0.828 |
| Family planning | 16 | 2.9 (1.7, 4.8) | 13 | 3.0 (1.7, 5.2) | 2 | 3.1 (0.5, 11.6) | 1.000 | 3 | 2.8 (0.7, 8.4) | 1.000 |
| Transport system | 15 | 2.7 (1.6, 4.6) | 14 | 3.2 (1.8, 5.4) | 1 | 1.5 (0.1, 9.4) | 0.705 | 1 | 0.9 (0.0, 5.7) | 0.324 |
| ***Deaths Among Young Children*** | | | | | | | | | | |
| **Deaths deemed to be preventable** |  |  |  |  |  |  | 0.025 |  |  | 0.013 |
| Yes | 716 | 89.2 (86.8, 91.2) | 625 | 90.3 (87.8, 92.4) | 48 | 80.0 (67.3, 88.8) |  | 91 | 82.0 (73.3, 88.4) |  |
| No | 87 | 10.8 (8.8, 13.2) | 67 | 9.7 (7.6, 12.2) | 12 | 20.0 (11.2, 32.7) |  | 20 | 18.0 (11.6, 26.7) |  |
| **Recommendations** |  |  |  |  |  |  |  |  |  |  |
| ANC/Obstetric Care | 18 | 2.7 (1.7, 4.3) | 14 | 2.4 (1.4, 4.1) | 3 | 6.8 (1.8, 19.7) | 0.109 | 4 | 5.0 (1.6, 13.0) | 0.256 |
| Clinical Management | 400 | 60.1 (56.2, 63.8) | 345 | 58.9 (54.8, 62.9) | 31 | 70.5 (54.6, 82.8) | 0.153 | 55 | 68.8 (57.3, 78.4) | 0.113 |
| Health-seeking Behavior | 320 | 48.0 (44.2, 51.9) | 281 | 48.0 (43.8, 52.1) | 20 | 45.5 (30.7, 61.0) | 0.758 | 39 | 48.8 (37.5, 60.1) | 0.906 |
| HIV prevention | 45 | 6.8 (5.0, 9.0) | 37 | 6.3 (4.5, 8.7) | 3 | 6.8 (1.8, 19.7) | 0.753 | 8 | 10.0 (4.7, 19.3) | 0.233 |
| Health Education | 332 | 49.8 (46.0, 53.7) | 298 | 50.9 (46.7, 55.0) | 19 | 43.2 (28.7, 58.9) | 0.351 | 34 | 42.5 (31.7, 54.0) | 0.190 |
| Nutritional Support | 176 | 26.4 (23.1, 30.0) | 145 | 24.7 (21.3, 28.5) | 14 | 31.8 (19.1, 47.7) | 0.286 | 31 | 38.8 (28.3, 50.3) | 0.010 |
| Infection prevention | 182 | 27.3 (24.0, 30.9) | 165 | 28.2 (24.6, 32.0) | 10 | 22.7 (12.0, 38.2) | 0.490 | 17 | 21.2 (13.2, 32.1) | 0.229 |
| Vaccinations | 46 | 6.9 (5.2, 9.2) | 33 | 5.6 (4.0, 7.9) | 10 | 22.7 (12.0, 38.2) | <0.001 | 13 | 16.2 (9.3, 26.6) | 0.002 |
| Family planning | 12 | 1.8 (1.0, 3.2) | 10 | 1.7 (0.9, 3.2) | 2 | 4.5 (0.8, 16.7) | 0.202 | 2 | 2.5 (0.4, 9.6) | 0.645 |
| Transport system | 23 | 3.5 (2.3, 5.2) | 16 | 2.7 (1.6, 4.5) | 4 | 9.1 (3.0, 22.6) | 0.044 | 7 | 8.8 (3.9, 17.7) | 0.014 |

*Recommendations for how to prevent deaths were available for 233 (89.3%) of the 261 preventable neonatal deaths.

**Recommendations for how to prevent deaths were available for 547 (87.9%) of the 622 preventable deaths among infants.

***Recommendations for how to prevent deaths were available for 666 (93.0%) of the 716 preventable deaths among children.
