# Supplementary material for: Deaths with preceding hospitalisations within 180 days in eight countries in sub-Saharan Africa and South Asia: A secondary descriptive analysis of the Child Health and Mortality Prevention Surveillance (CHAMPS) network
Source: BMJ Open. 2026 Mar 23;16(3):e106095. doi: 10.1136/bmjopen-2025-106095 (PMC13034342; doi:10.1136/bmjopen-2025-106095)
Supplement: Supplementary file 2 [file bmjopen-2025-106095.reviewer_comments.pdf]

PEER REVIEW HISTORY

BMJ Open publishes all reviews undertaken for accepted manuscripts. Reviewers are asked to complete a checklist review form and are provided with free text boxes to elaborate on their assessment. These free text comments are reproduced below.

ARTICLE DETAILS

Title (Provisional)

Deaths with Preceding Hospitalizations within 180 days in Eight Countries in sub-Saharan Africa and South Asia: A Secondary Descriptive Analysis of the Child Health and Mortality Prevention Surveillance (CHAMPS) Network

Authors

Varo, Rosauro; Cole, Kaitlin; Madewell, Zachary J.; Iglesias, Jaime Fanjul; Igunza, Kitiezo Aggrey; Akelo, Victor; Mugah, Christopher; Onyango, Dickens Otieno; Were, Joyce; Madhi, Shabir A; Dangor, Ziyaad; Johnstone, Siobhan; Lala, Sanjay; Ruder, Tanya; Mandomando, Inacio; Kincardett, Milton; Xerinda, Elisio G.; Scott, A; Assefa, Nega; Madrid-Castillo, Lola; Hassen, Faisel Abdi; Edris, Yunus; Ogbuanu, Ikechukwu; Bassey, Ima-Abasi; Samura, Solomon; Sillah, Abdul Salaam; Kaluma, Erick; Arifeen, Shams E.; Biswas, Rajib; Gurley, Emily S; Rahman, Afruna; Hossain, Mohammad Zahid; Omer, Saad B; Kazi, Abdul Momin; Belgaumi, Sameer M; Allana, Raheel; Keita, Adama M.; Bassat, Quique; Mutevedzi, Portia C.; Whitney, Cynthia G.; Rees, Chris; Network, CHAMPS

VERSION 1 - REVIEW

**Reviewer**	**1**
**Name**	**Kabakyenga, Jerome**
**Affiliation**	**Mbarara University of Science and Technology, Maternal, Newborn and Child health Institute**
**Date**	**22-Aug-2025**
**COI**	**None**

Athough the study focused on post discharge deaths the authors ought to have also reported more on the deaths without preceding hospitalizations as a comparison group.

**Reviewer**	**2**
**Name**	**Jayaratne, Kapila**
**Affiliation**	**University of Colombo Faculty of Medicine, Community Medicine**

**Date**	**24-Oct-2025**
**COI**	**None**

This multi-country descriptive analysis of post-discharge mortality of newborns, infants and children who had been hospitalized within the preceding 180 days contributes to filling the research gap on this topic with a rich data set spanning across eight countries with different contexts.

Suggest rewording the title to reflect the countries or regions where the CHAMPS Network was implemented.

A short overview of child mortality in countries of CHAMPS -Bangladesh, Ethiopia, Kenya, Mali, Mozambique, Pakistan, Sierra Leone, and South Africa - would give a valuable insight to the reader into the regional patterns and burden of child deaths.

Methods:

The working criterion "a hospitalization within the preceding 180 days" – is too long to be categorized as post-discharge mortality.

The Inclusion and Exclusion Criteria are confusing and not clearly articulated. It should clearly mention that deaths in live-born children aged 0–59 months who had an identifiable hospitalization and hospital discharge date ≤ 180 days prior to death were included in the study. However, this definition does not specify the nature of the hospitalization — whether it was for delivery (eg. live birth) or for a subsequent illness episode. It seems that some inclusion criteria are indicated in exclusions. This mixing of criteria creates ambiguity. The authors should explicitly define the inclusion criteria and separate methodological exclusions. This clarification is essential to ensure that the study truly captures post-discharge mortality.

The relatively small final subsample (n=733 post-discharge deaths) represents only a fraction of total child deaths and is unlikely to be representative of the wider CHAMPS mortality cohort. This small subsample limits statistical power for subgroup analysis, particularly when stratified by country or age category. Reported P-values may therefore be unstable and sensitive to small changes in categorization.

Add a short description of DeCoDe to help readers understand its composition, role, process in identifying and certifying causes of child deaths and esp on providing recommendations for preventability. It is not clear whether they adopted an objective framework or model to determine preventability of deaths.

Results:

Suggest presenting a more rational breakdown—preferably in a tabular format—of the time from hospital discharge to death to allow a clearer and more meaningful interpretation of

post-discharge mortality patterns. The figure 2 does not properly provide this information to the reader.

The results do not provide sufficient evidence or context to establish a clear association between the deaths and hospital discharge. Many of the reported causes or circumstances of death appear to be unrelated to the discharge event, which in turn dilutes the essence of the study's title. As such, I suggest changing the title to "outside hospital deaths" or similar terminology, rather than "post-discharge mortality."

According to the variables section, Prior hospitalizations are captured in CHAMPS as both discrete variables and in narrative fields summarizing both clinical data and verbal autopsies. This implies that the dataset does contain sufficient qualitative and quantitative information to link each death to the reason for prior hospitalization. However, in the limitations, the authors acknowledge that they were unable to capture reasons or durations for preceding hospitalizations to link those diagnoses to ultimate causes of post-discharge mortality. This appears inconsistent. If such data were available, it should have been possible to extract and code at least broad diagnostic categories. Therefore, the failure to analyze or present this linkage represents a major missed analytical opportunity.

The term "congenital birth defects" is used incorrectly, as it is redundant and has been repeated multiple times throughout the text. The correct terminology should be either "congenital abnormalities" or "birth defects."

The results section on the preventability of post-discharge mortality could be improved by providing clearer, objective and evidence-based recommendations. The recommendations listed in Table 2 appear vague and lacks clear criteria or rationale.

Findings do not implicate that suboptimal clinical care contributed to the burden of post-discharge mortality, even though the discussion section suggests such a link.

Given that two clinicians independently reviewed narrative and clinical summaries for each case, the authors could have gone further to extract qualitative indicators of suboptimal clinical care — such as missed diagnoses, delays in treatment, or premature discharges — to support their discussion points on suboptimal clinical care. Therefore, the authors had an opportunity to extract and thematically analyze these details to substantiate their discussion claim.

The leading causes of "post-discharge mortality" identified in this study include particularly malnutrition, prematurity complications, and congenital defects which are chronic or inherent conditions rather than outcomes directly influenced by the preceding hospitalization. There is no basis for assuming that the hospital care received was relevant to the cause of death. Drawing conclusions or formulating recommendations about hospital management or discharge practices based on these deaths is consequently not valid.

The main flaw of the study is that the authors did not capture the reasons for, or durations of, preceding hospitalizations—information essential to linking initial diagnoses with the

ultimate causes of post-discharge mortality. Without establishing clear links between the causes or circumstances of all deaths and the preceding hospital management, it seems unreasonable to analyze or discuss these cases under the name of “post-discharge mortality”.

**Reviewer**	**3**
**Name**	**Dack, Kyle**
**Affiliation**	**University of Bristol, MRC Integrative Epidemiology Unit**
**Date**	**22-Nov-2025**
**COI**	**None**

Statistical review summary:

The methods are clear, and the statistical analysis is reasonable. The study is descriptive and uses the CHAMPS dataset of deaths during early childhood. There is a risk using this database of unsuitable generalisations, or mis-using it to evaluate survival, but the authors avoid this and the language used throughout the manuscript is fair.

However, I recommend rephrasing certain elements of the results which I have quoted below to ensure full accuracy.

Abstract:

1. The study is described as descriptive, aiming to summarise patterns within this specific population. Given that inference is not a goal, I suggest clarifying the aims to reflect this – by replacing “determine” with “describe”. This seems like a minor change but the existing aim, particularly “determine causes of post-discharge mortality” is easy to misread as meaning some form of causal inference will be applied.

2. “Post-discharge mortality was most common in deaths among infants aged 1-11 months (P<0.001) and among those with weight-for-age Z-score of <-3 (P<0.001) compared to deaths in other age groups or less malnourished children” I do not think mortality is the most accurate term, as mortality was 100% for all groups. It would be more valid to report this as “The greatest proportion of deaths occurred between 1-11 months (48.0%), compared to 0-1 month (21.7%) and 1-5 years (30.3%).” Or similar. Additionally, the p-value was not a comparison between only these three groups, it was based on a chi-square test of post-discharge vs not post-discharge, so does not seem fully relevant to this comparison.

Introduction:

3. Aim 4 is “to identify risk factors associated with post-discharge mortality among cases”. Looking at the results, I don’t think this is performed? Risk factors are not mentioned anywhere in the methods or results. There is stratified analyses briefly reported in the results but this is never framed as identifying risk factors, instead it is all presented

descriptively, which aligns with the stated study design. I suggest clarifying or changing this so that it clearly links to the presented results.

Methods:

4. Study design – to help readers get a sense of the study I suggest briefly restating the date range in one of the first sentences.

5. Variables – To align with STROBE please report all variables which were of interest (e.g. remove the "etc"), including any transformations or categorizations applied for this particular study.

6. "time-to-event distributions for each age group " – what were these age groups?

7. "We used the chi-square test to identify subsets of child deaths with higher contributions to post-discharge mortality." Which subsets were compared?

Results

8. "The proportion of post-discharge mortality among enrolled deaths was 16.7%" Mortality was 100% because enrollment was based upon death. I can see that the authors are comparing to all the included data but the phrasing does not make this clear. I suggest rephrasing, e.g. Out of the 4,378 total deaths, 16.7% were deaths that occurred within 6 months of a hospitalization. This removes the "mortality" phrase which has a risk of being misinterpreted.

9. "Among post-discharge deaths, 159 (21.7%), 352 (48.0%) and 222 (30.3%) were neonates, infants and children, respectively" I suggest adding the age ranges in-text so that readers can interpret without checking the table.

10. "Post-discharge mortality was more common among infants than neonates and young children (P<0.001; Table 1)."

-Similar to the above comment - mortality was 100% in all groups, as this is a cohort of deaths only. It should not be possible to determine mortality rates because there is no denominator, i.e. no survivors are present. Table 1 shows that among those who died, most deaths occurred during infancy. I recommend rephrasing to ensure this is not misinterpreted. Additionally, the p-value referenced was not comparing the distribution of age groups in post-discharge deaths – it compared among those who were and were not hospitalized. I recommend adding this information or dropping the p-value (as this is descriptive I do not think the p-values are essential).

11. "The proportion of deaths with prior hospitalizations varied by site. Post-discharge mortality was most common among deaths among cases with weight-for-age Z-score of <-3 compared to less malnourished children (P<0.001)." The above comments also apply to these sentences which I recommend rephrasing to avoid readers misinterpreting it as mortality rates, and adding information about the actual % difference if possible.

12. "Among post-discharge mortality cases, the AFT model estimated the median time (50% survival) from discharge to death as 24 days" I suggest avoiding the term survival as no formal survival analysis could be conducted, as there are no survivors to include. I suggest rephrasing or removing the 50% term as the sentence would be clearer without it.

13. Figures and tables: No suggestions or comments – all are clear and consistent.

Discussion:

14. The discussion is supported by the data, and gives a balanced and critical appraisal of the findings.

VERSION 1 - AUTHOR RESPONSE

Reviewer: 1, Dr. Jerome Kabakyenga, Mbarara University of Science and Technology
Comments to the Author:
Although the study focused on post discharge deaths the authors ought to have also reported more on the deaths without preceding hospitalizations as a comparison group.
Author response: We appreciate the time the Reviewer spent providing their input on our manuscript. We have included the following comparisons of cases of post-discharge mortality to cases without preceding hospitalizations.

-Figure S2: Immediate causes of death compared between cases of post-discharge mortality to cases without preceding hospitalizations
-Figure S3: Underlying causes of death compared between cases of post-discharge mortality to cases without preceding hospitalizations
-Figure S4: Co-morbid causes of death compared between cases of post-discharge mortality to cases without preceding hospitalizations
-Table 2: Comparison of preventability of death between cases of post-discharge mortality to cases without preceding hospitalizations
-Table S2: Comparison of preventability of death between cases of post-discharge mortality to cases without preceding hospitalizations by age

The specifics of these results are reported throughout the Results section of the manuscript.

Reviewer: 2, Dr. Kapila Jayaratne, University of Colombo Faculty of Medicine
Comments to the Author:
This multi-country descriptive analysis of post-discharge mortality of newborns, infants and children who had been hospitalized within the preceding 180 days contributes to filling the research gap on this topic with a rich data set spanning across eight countries with different contexts.
Author response: We appreciate the Reviewer's summary of our findings. We also appreciate their suggestions, which we have incorporated as outlined below.

Suggest rewording the title to reflect the countries or regions where the CHAMPS Network was implemented.
Author response: We have changed the title to read, "*Post-Discharge Mortality in Eight Countries in sub-Saharan Africa and South Asia: A Secondary Descriptive Analysis of the Child Health and Mortality Prevention Surveillance (CHAMPS) Network*".

A short overview of child mortality in countries of CHAMPS -Bangladesh, Ethiopia, Kenya, Mali, Mozambique, Pakistan, Sierra Leone, and South Africa - would give a valuable insight to the reader into the regional patterns and burden of child deaths.
Author response: We have added the following under Study Setting in response to the Reviewer's

comment, “These sites were selected for CHAMPS because they had mortality rates among children aged <5 years of >50/1,000 live births at the time that implementation activities of CHAMPS began. These sites are in countries which continue to experience a high burden of mortality among children aged <5 years, ranging from 30.6 deaths/1,000 live births in Bangladesh to 94.3 deaths/1,000 live births in Sierra Leone.” As outlined in the table below from the World Bank (https://data.worldbank.org/indicator/SH.DYN.MORT).

	**Under 5 Mortality Rates (Deaths/1,000 Live Births) in 2023**
Bangladesh	30.6
Ethiopia	46.5
Kenya	39.9
Mali	91.3
Mozambique	61.7
Pakistan	58.5
Sierra Leone	94.3
South Africa	34.7

Methods:
The working criterion “a hospitalization within the preceding 180 days” – is too long to be categorized as post-discharge mortality.
Author response: We appreciate the Reviewer’s comment. Indeed, this is a topic of much debate in the literature as some studies have used 60 days, others have used 90 days, and the majority have used 180 days as summarized in our recent systematic review (i.e., Ginjupalli R, et al. PLOS Glob Public Health. 2025. and Knappett M, et al. EClinicalMedicine. 2023.). At this point, it is unclear what the optimal follow up timeframe is. In order to address the Reviewer’s concern, we have included a sensitivity analysis restricting the follow up to 90 days following hospital discharge and included these results in Table 2, Table S1, Table S2, and Figure 3B.

The Inclusion and Exclusion Criteria are confusing and not clearly articulated. It should clearly mention that deaths in live-born children aged 0–59 months who had an identifiable hospitalization and hospital discharge date ≤ 180 days prior to death were included in the study. However, this definition does not specify the nature of the hospitalization — whether it was for delivery (eg. live birth) or for a subsequent illness episode. It seems that some inclusion criteria are indicated in exclusions. This mixing of criteria creates ambiguity. The authors should explicitly define the inclusion criteria and separate methodological exclusions. This clarification is essential to ensure that the study truly captures post-discharge mortality.
Author response: We have clarified the language in this section in response to the Reviewer’s comment. Specifically, we have removed mention of “liveborn children” because CHAMPS enrolls cases of mortality after the time of death. Additionally, we have clarified the inclusion criteria for these analyses as follows, “We included deaths enrolled in CHAMPS from 2016-2023 aged 0-59 months at the time of death who underwent full postmortem examination and those with verbal autopsies that allowed for the identification of previous hospitalizations.”

Regarding the nature of the hospitalizations, we have added a paragraph to clarify what we included as hospitalizations. “When identifying prior hospitalizations, we did not classify hospitalizations for delivery as a prior hospitalization if the child did not receive any care beyond routine newborn care. We did, however, include any hospitalizations for delivery where newborns required additional interventions as well as any hospitalizations for illness episodes.”

Regarding reasons for hospital admission, we have included the following in the Limitations paragraph to note that we did not have access to reasons for index hospitalizations that preceded mortality, “Moreover, we were unable to capture reasons or durations for preceding hospitalizations to link those diagnoses to ultimate causes of post-discharge mortality.”

The relatively small final subsample (n=733 post-discharge deaths) represents only a fraction of total child deaths and is unlikely to be representative of the wider CHAMPS mortality cohort. This small subsample limits statistical power for subgroup analysis, particularly when stratified by country or age category. Reported P-values may therefore be unstable and sensitive to small changes in categorization.
Author response: We appreciate the Reviewer's concern and we understand we should be cautious in the generalization of the results. However, there were 733 cases of post-discharge mortality, which represents 16.7% of cases that met inclusion criteria (i.e., were eligible to have an antecedent hospitalization, n=4,378). Additionally, this is the largest sample of cases of post-discharge mortality in the current literature, and we think this a strength of our analysis that should be taken into account.

Regarding the potential instability of *P* values with the subgroup analyses, we believe, as said above, that the number of cases we have included in the analysis is large enough to balance any potential instability. Having said that, we have added the following to the Limitations in response to the Reviewer's comment: "Some of our subgroup analyses by age or by CHAMPS site may lead to potential instability of significance due to the diminution of number of cases. Thus, future single-center studies may be indicated to better understand causes of post-discharge mortality by CHAMPS site with larger sample sizes."

Add a short description of DeCoDe to help readers understand its composition, role, process in identifying and certifying causes of child deaths and esp on providing recommendations for preventability. It is not clear whether they adopted an objective framework or model to determine preventability of deaths.
Author response: We appreciate the opportunity to provide additional clarity on the DeCoDe process. In the third paragraph under Data Sources in the Methods, we have added the following, "In addition to assigning causes of death, DeCoDe panels determine whether each death was preventable and, if deemed preventable through consensus, specifically how each death could have been averted. This is done by selecting from a list of personal and health systems approaches that could be implemented to avert childhood deaths. This list of health systems improvements was developed iteratively by DeCoDe panels at each site when CHAMPS began in 2016."

Results:
Suggest presenting a more rational breakdown—preferably in a tabular format—of the time from hospital discharge to death to allow a clearer and more meaningful interpretation of post-discharge mortality patterns. The figure 2 does not properly provide this information to the reader.
Author response: We have added the following to the first paragraph under Timing of deaths after hospital discharge as requested, "Among post-discharge mortality cases, 74.2% (n=544/733) had available dates for both hospital discharge and death. Among these, 52.4% (n=285/544) of post-discharge deaths occurred 1–30 days after discharge, 18.2% (n=99/544) occurred 31–60 days, 11.9% (n=65/544) occurred 61–90 days, and 17.5% (n=95/544) occurred 91–180 days following discharge."

The results do not provide sufficient evidence or context to establish a clear association between the deaths and hospital discharge. Many of the reported causes or circumstances of death appear to be unrelated to the discharge event, which in turn dilutes the essence of the study's title. As such, I suggest changing the title to "outside hospital deaths" or similar terminology, rather than "post-discharge mortality."
Author response: We thank the Reviewer for this opportunity to provide additional clarity. Indeed, we were not able to establish a clear association between the reason for hospitalization during antecedent hospitalizations and death because reasons for prior hospitalizations were not always reported by caregivers in verbal autopsies. This is noted in the Limitations as above. That said, many families of the deceased noted prior hospitalizations (though not always the reason for those prior hospitalizations), which is what allowed for these important and novel analyses on cause of death in this population. As only 55.7% of cases of post-discharge mortality occurred outside of healthcare

facilities (meaning, 44.3% occurred in healthcare facilities), we would ask to retain the title as is. Finally, we have added the following important line to the Conclusions to emphasize the Reviewer's point: "Moreover, additional studies are warranted to assess the potential association between specific reasons for hospitalization in index hospitalizations and postmortem determined causes of death to further inform interventions to reduce potentially preventable cases of post-discharge mortality."

According to the variables section, Prior hospitalizations are captured in CHAMPS as both discrete variables and in narrative fields summarizing both clinical data and verbal autopsies. This implies that the dataset does contain sufficient qualitative and quantitative information to link each death to the reason for prior hospitalization. However, in the limitations, the authors acknowledge that they were unable to capture reasons or durations for preceding hospitalizations to link those diagnoses to ultimate causes of post-discharge mortality. This appears inconsistent. If such data were available, it should have been possible to extract and code at least broad diagnostic categories. Therefore, the failure to analyze or present this linkage represents a major missed analytical opportunity.
Author response: We attempted to extract reasons and durations for antecedent hospitalizations as we reviewed both quantitative and qualitative data fields. Many families of the deceased noted prior hospitalizations (though not always the reason or duration for those prior hospitalizations), which is what allowed for these important and novel analyses on cause of death in this population. In response to the Reviewer's comment, we have added the following to the Methods under Variables, "We also attempted to capture reasons for antecedent hospitalizations and duration of those hospitalizations. However, we were unable to reliably extract this information from the narrative fields in verbal autopsies, in which families are asked about meticulous details on the events around the time of death, but may not capture historical details such as reasons for or durations of antecedent hospitalizations."

Furthermore, we have noted this in the Limitations in the following sentence, "Moreover, we were unable to capture reasons or durations for preceding hospitalizations to link those diagnoses to ultimate causes of post-discharge mortality."

Finally, as explained in the point above, we have added the following to the Conclusions in response to this comment: "Moreover, additional studies are warranted to assess the potential association between specific reasons for hospitalization in index hospitalizations and postmortem determined causes of death to further inform interventions to reduce potentially preventable cases of post-discharge mortality."

The term "congenital birth defects" is used incorrectly, as it is redundant and has been repeated multiple times throughout the text. The correct terminology should be either "congenital abnormalities" or "birth defects."
Author response: We appreciate the Reviewer's comment. In response, we have changed all mention of "congenital birth defects" to "birth defects" as recommended.

The results section on the preventability of post-discharge mortality could be improved by providing clearer, objective and evidence-based recommendations. The recommendations listed in Table 2 appear vague and lacks clear criteria or rationale.
Findings do not implicate that suboptimal clinical care contributed to the burden of post-discharge mortality, even though the discussion section suggests such a link.
Author response: We appreciate the Reviewer's comment. In terms of evidence-based recommendations, these preventability categories were developed in CHAMPS as there were no such standards. Several papers have been published using these standards which have also been systematically applied to all CHAMPS studies and provided valuable and specific information in relation to prevention of children deaths (i.e., Madewell, et al. JAMA Network Open. 2022. and Rahman A, et al. JAMA Network Open. 2025). The process of developing these preventability categories, the description of this process has been added to the Methods under Data Sources as follows, "In addition to assigning causes of death, DeCoDe panels determine whether each death was preventable and, if deemed preventable through consensus, specifically how each death could have

been averted. This is done by selecting from a list of personal, clinical and health systems approaches that could be implemented to avert childhood deaths. This list of health systems improvements was developed iteratively by DeCoDe panels at each site when CHAMPS began in 2016."

In terms of the Reviewer's concern about the reporting of a link between suboptimal clinical care and the burden of post-discharge mortality, we have added the following to the Discussion to more fully discuss this important point, "It is unclear if DeCoDe panels were referring to the clinical care provided in antecedent hospitalizations or that around the time of death for cases of post-discharge mortality. However, given that cases of post-discharge mortality occurred following hospitalization, it is possible that DeCoDe panelists considered the same when making this recommendation."

Given that two clinicians independently reviewed narrative and clinical summaries for each case, the authors could have gone further to extract qualitative indicators of suboptimal clinical care — such as missed diagnoses, delays in treatment, or premature discharges — to support their discussion points on suboptimal clinical care. Therefore, the authors had an opportunity to extract and thematically analyze these details to substantiate their discussion claim.
Author response: We appreciate this comment. As above, information on antecedent hospitalizations came from both clinical notes as well as families retelling of the events surrounding death. As such, information on specific elements of clinical care that were suboptimal were not always recorded. We have added the following to the paragraph in the Discussion on preventability to address this concern, "Ultimately, future prospective studies that are designed to fully assess the quality of clinical care during index hospitalizations coupled with patient follow up are warranted to better link suboptimal clinical care to post-discharge mortality."

The leading causes of "post-discharge mortality" identified in this study include particularly malnutrition, prematurity complications, and congenital defects which are chronic or inherent conditions rather than outcomes directly influenced by the preceding hospitalization. There is no basis for assuming that the hospital care received was relevant to the cause of death. Drawing conclusions or formulating recommendations about hospital management or discharge practices based on these deaths is consequently not valid.
Author response: We have added the following to the final paragraph of the Discussion in response to the Reviewer's insight, "Furthermore, as many of the causes of death among cases of post-discharge mortality were due to chronic conditions, it is essential to improve the continuum of clinical care during the hospitalization but also during follow-up, in order to prevent cases of post-discharge mortality."

The main flaw of the study is that the authors did not capture the reasons for, or durations of, preceding hospitalizations—information essential to linking initial diagnoses with the ultimate causes of post-discharge mortality. Without establishing clear links between the causes or circumstances of all deaths and the preceding hospital management, it seems unreasonable to analyze or discuss these cases under the name of "post-discharge mortality".
Author response: We appreciate the perspective of this Reviewer. The main contribution of our analysis is that, with the methods of CHAMPS, we are giving a clear insight in the specific causes of death in cases of PDM, which until now have not been explored. This is the reason we think that our manuscript fills a key gap in the literature by providing definitive postmortem determined causes of death as well as by describing the overall proportion of deaths that enroll in active surveillance that had antecedent hospitalizations. There are no other studies that are able to answer these questions the same way that CHAMPS can. We hope that our responses to each comment above, including the comments reiterated here, help convey the importance of these findings and provide clarity on the reporting. Again, we appreciate the Reviewer's input and we are certain that the incorporation of their input has greatly strengthened the reporting of our work.

Reviewer: 3, Dr. Kyle Dack, University of Bristol

Comments to the Author:
Statistical review summary:
The methods are clear, and the statistical analysis is reasonable. The study is descriptive and uses the CHAMPS dataset of deaths during early childhood. There is a risk using this database of unsuitable generalisations, or mis-using it to evaluate survival, but the authors avoid this and the language used throughout the manuscript is fair.
However, I recommend rephrasing certain elements of the results which I have quoted below to ensure full accuracy.
Author response: We thank the Statistical Reviewer for their overall summary provided here. We have responded to each comment below and incorporated the Statistical Reviewer's input as described under each point.

Abstract:
1. The study is described as descriptive, aiming to summarise patterns within this specific population. Given that inference is not a goal, I suggest clarifying the aims to reflect this – by replacing "determine" with "describe". This seems like a minor change but the existing aim, particularly "determine causes of post-discharge mortality" is easy to misread as meaning some form of causal inference will be applied.
Author response: We thank the Reviewer for this suggestion. "Determine" has been changed to "describe" both in the Abstract as well as in the Introduction where the Objectives are laid out.

2. "Post-discharge mortality was most common in deaths among infants aged 1-11 months (P<0.001) and among those with weight-for-age Z-score of <-3 (P<0.001) compared to deaths in other age groups or less malnourished children" I do not think mortality is the most accurate term, as mortality was 100% for all groups. It would be more valid to report this as "The greatest proportion of deaths occurred between 1-11 months (48.0%), compared to 0-1 month (21.7%) and 1-5 years (30.3%)." Or similar. Additionally, the p-value was not a comparison between only these three groups, it was based on a chi-square test of post-discharge vs not post-discharge, so does not seem fully relevant to this comparison.
Author response: We have changed this text as recommended in the Abstract and in the Results to read as follows, "The greatest proportion of deaths with antecedent hospital admissions occurred among cases aged 1-11 months (48.0%), compared to those aged 0-1 months (21.7%) and those aged 1-5 years (30.3%). Moreover, the greatest proportion of deaths with antecedent hospital admissions occurred among children with weight-for-age Z-score of <-3 (28.4%) compared to those with weight-for-age Z-score of >-2 and those with weight-for-age Z-score between -2 and -3 (11.7%)."

Introduction:
3. Aim 4 is "to identify risk factors associated with post-discharge mortality among cases". Looking at the results, I don't think this is performed? Risk factors are not mentioned anywhere in the methods or results. There is stratified analyses briefly reported in the results but this is never framed as identifying risk factors, instead it is all presented descriptively, which aligns with the stated study design. I suggest clarifying or changing this so that it clearly links to the presented results.
Author response: We appreciate the opportunity to provide clarifying language in our Aims. They have been changed in the final paragraph of the Introduction to read, "Here, our objective was to measure the contribution of post-discharge mortality to all deaths in children aged 0-59 months, assess the timing of post-discharge mortality following hospital discharge, describe causes of post-discharge mortality, and to identify populations of child deaths who most commonly had antecedent hospital admissions in the eight-country Child Health and Mortality Prevention Surveillance (CHAMPS) network."

Methods:
4. Study design – to help readers get a sense of the study I suggest briefly restating the date range in one of the first sentences.
Author response: We have added the date range to the first sentence of the Study Design section of

the Methods as follows, “We conducted a descriptive study using data that were collected as part of prospective childhood mortality surveillance in the CHAMPS network from 2016-2023.”

5. Variables – To align with STROBE please report all variables which were of interest (e.g. remove the “etc”), including any transformations or categorizations applied for this particular study.
Author response: We have added the other variables as requested as included in this sentence that appears under Variables in the Methods, “For this study, we analyzed discrete CHAMPS variables (e.g., age at the time of death, sex, site of death, place of death, immediate cause of death, underlying cause of death, breastfeeding status, weight-for-age Z score, whether the case underwent full postmortem testing, and whether the death was preventable and how it may have been preventable).”

6. “time-to-event distributions for each age group “ – what were these age groups?
Author response: We have added these age groups in the text under Statistical Analyses as follows, “Time to event analysis was conducted using Kaplan Meier Curves demonstrating time from hospital discharge to death and Weibull Accelerated Failure Time (AFT) models to estimate time-to-event distributions for each age group at the time of hospital admission (i.e., neonates aged 0-27 days at death, infants aged 1-11 months at death, and young children aged 12-59 months at death).”

7. “We used the chi-square test to identify subsets of child deaths with higher contributions to post-discharge mortality.” Which subsets were compared?
Author response: We thank the Reviewer for this recommendation. In response, we have changed the text in the final paragraph of the Methods to read, “We used the chi-square test to identify subsets of child deaths (i.e., age group at the time of death, sex, site of death, place of death, breastfeeding status, weight-for-age Z score, whether the case underwent full postmortem testing, and whether the death was preventable and how it may have been preventable) with higher proportions of post-discharge mortality.”

Results
8. “The proportion of post-discharge mortality among enrolled deaths was 16.7%” Mortality was 100% because enrollment was based upon death. I can see that the authors are comparing to all the included data but the phrasing does not make this clear. I suggest rephrasing, e.g. Out of the 4,378 total deaths, 16.7% were deaths that occurred within 6 months of a hospitalization. This removes the “mortality” phrase which has a risk of being misinterpreted.
Author response: We have changed this text both in the Abstract and in the Results as recommended with the following language, “Out of the 4,378 total deaths, 16.7% were deaths that occurred within 6 months of a hospitalization (n=733/4,378).”

9. “Among post-discharge deaths, 159 (21.7%), 352 (48.0%) and 222 (30.3%) were neonates, infants and children, respectively” I suggest adding the age ranges in-text so that readers can interpret without checking the table.
Author response: We have updated the text as recommended. This now reads, “Among post-discharge deaths, 159 (21.7%, 95% CI: 18.8%–24.9%) were neonates aged 0–27 days at the index hospitalization, 352 (48.0%, 95% CI: 44.4%–51.7%) were infants aged 28-365 days at the index hospitalization, and 222 (30.3%, 95% CI: 27.0%–33.8%) were children aged 12-59 months at the index hospitalization (Table 1).”

10. “Post-discharge mortality was more common among infants than neonates and young children (P<0.001; Table 1).”
-Similar to the above comment - mortality was 100% in all groups, as this is a cohort of deaths only. It should not be possible to determine mortality rates because there is no denominator, i.e. no survivors are present. Table 1 shows that among those who died, most deaths occurred during infancy. I recommend rephrasing to ensure this is not misinterpreted. Additionally, the p-value referenced was not comparing the distribution of age groups in post-discharge deaths – it compared among those who were and were not hospitalized. I recommend adding this

information or dropping the p-value (as this is descriptive I do not think the p-values are essential).
Author response: We appreciate the Reviewer's comment. We have rephrased this in the first paragraph of the Results as follows, "The greatest proportion of deaths with antecedent hospital admissions occurred among cases aged 1-11 months (48.0%), compared to those aged 0-1 months (21.7%) and those aged 1-5 years (30.3%; Table 1)." If the Editor allows, with the change in wording, we would request that the P values are retained.

11. "The proportion of deaths with prior hospitalizations varied by site. Post-discharge mortality was most common among deaths among cases with weight-for-age Z-score of <-3 compared to less malnourished children (P<0.001)." The above comments also apply to these sentences which I recommend rephrasing to avoid readers misinterpreting it as mortality rates, and adding information about the actual % difference if possible.
Author response: We have changed this text as recommended. This sentence in paragraph 1 of the Results now reads, "Moreover, the greatest proportion of deaths with antecedent hospital admissions occurred among children with weight-for-age Z-score of <-3 (28.4%) compared to those with weight-for-age Z-score of >-2 and those with weight-for-age Z-score between -2 and -3 (11.7%)."

12. "Among post-discharge mortality cases, the AFT model estimated the median time (50% survival) from discharge to death as 24 days" I suggest avoiding the term survival as no formal survival analysis could be conducted, as there are no survivors to include. I suggest rephrasing or removing the 50% term as the sentence would be clearer without it.
Author response: We have changed the text as recommended to remove any mention of survival from the Results.

13. Figures and tables: No suggestions or comments – all are clear and consistent.
Author response: We appreciate the Reviewer's assessment.

Discussion:
14. The discussion is supported by the data, and gives a balanced and critical appraisal of the findings.
Author response: We appreciate the Reviewer's assessment.

VERSION 2 - REVIEW

**Reviewer**	**2**
**Name**	**Jayaratne, Kapila**
**Affiliation**	**University of Colombo Faculty of Medicine, Community Medicine**
**Date**	**14-Jan-2026**
**COI**	

The authors have engaged constructively with the review comments and have made several important clarifications and textual improvements, including:

• Re-framing the title to reflect geography.

• Adding country-level under-five mortality context.

• Clarifying inclusion/exclusion criteria.

• Expanding the description of the DeCoDe process.

• Adding a stratified breakdown of time from discharge to death.

• Correcting terminology (“birth defects”).

• Explicitly acknowledging inability to link index admission diagnoses to death.

However, while editorial clarity has improved, the study's fundamental conceptual and causal limitations remain unresolved. The revision improves transparency but does not correct the core methodological constraint that dilutes the construct validity of “post-discharge mortality”.

Major Points

1. Conceptual Validity of “Post-Discharge Mortality”

The revised manuscript now explicitly acknowledges that:

“…we were unable to capture reasons or durations for preceding hospitalizations to link those diagnoses to ultimate causes of post-discharge mortality.”

This admission confirms that no causal or clinical linkage can be demonstrated between the index hospitalization and subsequent death. Therefore:

• The analytic construct remains temporal rather than etiologic.

• The category still mixes chronic/community deaths (malnutrition, prematurity complications, congenital defects) with discharge-related vulnerability.

Although the authors added statements in the Discussion and Conclusions calling for future linkage studies, this does not resolve the current misclassification problem. The term “post-discharge mortality” continues to imply hospital-linked vulnerability that is not empirically demonstrated.

As such, the current title continues to imply a causal and conceptual linkage between hospital discharge and subsequent deaths that is not supported by the available data. As the study is unable to establish the reason, duration, or clinical relevance of the index hospitalization in relation to the ultimate cause of death, the term “post-discharge mortality” is not methodologically defensible in its present usage. The analysis essentially describes deaths occurring within a defined time period after any prior hospitalization, rather than deaths attributable to discharge-related vulnerability or hospital care.

I therefore strongly recommend revising the title to reflect a temporally defined but etiologically neutral construct, such as “Deaths occurring within 180 days of prior hospitalization” or “Outside-hospital deaths following recent hospitalization”, to avoid causal over-interpretation and to accurately represent the analytical scope of the study.

2. Nature of Preceding Hospitalization (Delivery vs Illness)

The clarification that routine delivery admissions were excluded, while “delivery with additional interventions” and illness admissions were included is an improvement.

However, no results are presented distinguishing delivery-related vs illness-related index admissions. Thus, the dataset still likely contains etiologically heterogeneous deaths, which cannot be meaningfully interpreted under a single “post-discharge” concept.

3. Preventability and Suboptimal Clinical Care

The expanded DeCoDe description improves methodological transparency. However, preventability remains consensus-based without objective clinical linkage to index admissions. The authors explicitly state uncertainty whether “suboptimal clinical care” refers to care during hospitalization or at time of death. This undermines the validity of the claim that suboptimal hospital care contributes to post-discharge mortality burden. Thus, the main inferential concern remains partially but not substantively resolved.

4. Sample Derivation and Representativeness

The authors also acknowledge potential instability of subgroup analyses and have added this to Limitations. However, the analytical cohort still represents only 733 post-discharge deaths. This continues to constrain generalizability and causal interpretation, despite being “the largest dataset available”.

I appreciate the substantive revisions undertaken by the authors to improve the clarity, transparency, and reporting quality of the manuscript. However, significant conceptual and methodological limitations remain. A revision is therefore required to realign the manuscript.

VERSION 2 - AUTHOR RESPONSE

Reviewer 2 (Dr. Kapila Jayaratne, University of Colombo Faculty of Medicine)

The authors have engaged constructively with the review comments and have made several important clarifications and textual improvements, including:
• Re-framing the title to reflect geography.
• Adding country-level under-five mortality context.
• Clarifying inclusion/exclusion criteria.
• Expanding the description of the DeCoDe process.
• Adding a stratified breakdown of time from discharge to death.
• Correcting terminology (“birth defects”).
• Explicitly acknowledging inability to link index admission diagnoses to death.
Author response: We appreciate the Reviewer’s input and acknowledge their thoughtful input on this second round of revisions. We have replied to each new comment below and we are confident that our manuscript has been greatly strengthened thanks to their comments.

However, while editorial clarity has improved, the study's fundamental conceptual and causal limitations remain unresolved. The revision improves transparency but does not correct the core methodological constraint that dilutes the construct validity of “post-discharge mortality”.
Author response: We appreciate the Reviewer’s comment and we would like to highlight that we did not intended in any case to imply causality between what was going on with the cases we enrolled for postmortem testing during their prior hospitalization and discharge. We have removed the term “post-discharge mortality” and changed it to “deaths with preceding hospitalizations within 180 days” throughout. We believe that this

change should resolve any concerns about potential causality of the antecedent hospitalization and the postmortem-determined causes of death we describe.

Major Points
1. Conceptual Validity of "Post-Discharge Mortality"
The revised manuscript now explicitly acknowledges that:
"…we were unable to capture reasons or durations for preceding hospitalizations to link those diagnoses to ultimate causes of post-discharge mortality." This admission confirms that no causal or clinical linkage can be demonstrated between the index hospitalization and subsequent death. Therefore:
• The analytic construct remains temporal rather than etiologic.
Author response: We appreciate the Reviewer's comment. In response, as above, we have removed all mention of the terminology of "post-discharge mortality" to avoid any allusion to an attempt to link the causes of death that we describe with what was happening during the prior hospitalization. We hope that the complete removal of the phrase "post-discharge" will help avoid any potential interpretation that we were trying to link the occurrences of the preceding hospital admission with the death.

We have also made our objectives more explicit to focus on the temporal relationship we observe, as opposed to any etiologic relationship between antecedent hospitalizations and the postmortem-determined causes of death we describe. Due to the input from the Reviewer, our objectives now read, "Here, our objectives were to describe the frequency and timing of antecedent hospitalizations within 180 days among childhood deaths, identify populations of child deaths who most commonly had antecedent hospitalizations, describe causes of mortality among children with preceding hospitalizations within 180 days of death, and describe expert-panel public health recommendations on ways that future similar deaths could be averted in the eight-country Child Health and Mortality Prevention Surveillance (CHAMPS) network." Please note that this has not changed our analyses, but rather the way in which this information has been presented and interpreted.

• The category still mixes chronic/community deaths (malnutrition, prematurity complications, congenital defects) with discharge-related vulnerability.
Author response: We believe that the removal of any language that could be construed to imply causality between the reasons for prior hospitalizations and the deaths evaluated here has adequately shifted the focus to the frequency of antecedent hospitalizations among children who died in the 8 countries evaluated here. Instead, we are simply describing how common antecedent hospitalizations are among young children that died, when deaths occur among cases relative to their antecedent hospitalizations, postmortem-determined causes of death among such cases that had antecedent hospitalizations (without causal inference to what was happening during the preceding hospitalization), and expert-panel public health recommendations to potentially avert future similar deaths. We think that our major revisions throughout the manuscript illustrate this.

Although the authors added statements in the Discussion and Conclusions calling for future linkage studies, this does not resolve the current misclassification problem. The term "post-discharge mortality" continues to imply hospital-linked vulnerability that is not empirically demonstrated.
Author response: As mentioned above, we have removed all mention of the phrase "post-discharge mortality" to avoid any language that could be construed to imply causality between the reasons for prior hospitalizations and the deaths evaluated here. Furthermore, we have clarified our message in the Conclusions to read as follows, "Moreover, additional studies are warranted to assess the potential association between specific reasons for hospitalization in index hospitalizations and postmortem determined causes of death to further inform interventions to reduce potentially preventable future cases of mortality among children with preceding hospitalizations." With this, we want to avoid any allusion to a claim herein of causality between index hospitalizations and what we learned through extensive postmortem testing and documentation of the frequency of preceding hospitalizations.

As such, the current title continues to imply a causal and conceptual linkage between hospital discharge and subsequent deaths that is not supported by the available data. As the study is unable to establish the reason, duration, or clinical relevance of the index hospitalization in relation to the ultimate cause of death, the term "post-discharge mortality" is not methodologically defensible in its present usage. The analysis essentially describes deaths occurring within a defined time period after any prior hospitalization, rather than deaths attributable to discharge-related vulnerability or hospital care. I therefore strongly recommend revising the title to reflect a temporally defined but etiologically neutral construct, such as "Deaths occurring within 180 days of prior hospitalization" or "Outside-hospital deaths following recent hospitalization", to avoid causal over-interpretation and to accurately represent the analytical scope of the study.

Author response: As suggested, we have revised the title to now reads, “Deaths with Preceding Hospitalizations within 180 days in Eight Countries in sub-Saharan Africa and South Asia: A Secondary Descriptive Analysis of the Child Health and Mortality Prevention Surveillance (CHAMPS) Network”.

2. Nature of Preceding Hospitalization (Delivery vs Illness)
The clarification that routine delivery admissions were excluded, while “delivery with additional interventions” and illness admissions were included is an improvement.
However, no results are presented distinguishing delivery-related vs illness-related index admissions. Thus, the dataset still likely contains etiologically heterogeneous deaths, which cannot be meaningfully interpreted under a single “post-discharge” concept.
Author response: We appreciate the Reviewer’s concern. In response, we have added the following to the Methods under the Inclusion and Exclusion criteria, “However, we were unable to decipher specific reasons for delivery-related illnesses that resulted in hospitalization.”

Moreover, we have added the following to the Limitations paragraph, “We were also unable to distinguish between delivery-related hospitalizations for neonates and acute illnesses for neonates as we could not determine reasons for antecedent hospitalizations.”

Despite this, the aim of our study continues to be the same: to describe how common antecedent hospitalizations are in cases of childhood mortality and to describe postmortem-determined causes of death in cases that had preceding hospitalizations, which we have accomplished as outlined and have made clearer in our manuscript through extensive and major revisions in our resubmission.

3. Preventability and Suboptimal Clinical Care
The expanded DeCoDe description improves methodological transparency. However, preventability remains consensus-based without objective clinical linkage to index admissions. The authors explicitly state uncertainty whether “suboptimal clinical care” refers to care during hospitalization or at time of death. This undermines the validity of the claim that suboptimal hospital care contributes to post-discharge mortality burden. Thus, the main inferential concern remains partially but not substantively resolved.
Author response: We believe that the complete removal of the phrase “post-discharge mortality” sufficiently removes any opportunity to construe causal claims. Our intent with this objective was to describe panel recommendations to inform public health recommendations for the potential prevention of future deaths. We have included the following to avoid any interpretation of causality in the Methods under Data Sources, “In addition to assigning causes of death, based on what was learned about each case, DeCoDe panels provide expert-level, consensus opinion on public health interventions that could be employed to avoid future similar deaths. This is done by selecting from a list of personal and health systems approaches that could be implemented to avert future childhood deaths. These recommendations do not imply absolute preventability but rather are meant to inform future efforts to reduce future childhood deaths based on expert opinion.” As outlined above, we have also clarified that these are public health recommendations based on lessons learned to potentially avoid future deaths in the Results.

We have also added the following to the Discussion to call attention to the need to conduct prospective studies in the future, “Ultimately, future prospective studies that are designed to fully assess the quality of clinical care during index hospitalizations coupled with patient follow up are warranted to better link suboptimal clinical care to mortality following hospital discharge.”

4. Sample Derivation and Representativeness
The authors also acknowledge potential instability of subgroup analyses and have added this to Limitations. However, the analytical cohort still represents only 733 post-discharge deaths. This continues to constrain generalizability and causal interpretation, despite being “the largest dataset available”.
Author response: We have added the following to the Limitations paragraph in response to the Reviewer’s comment, “Although deaths included in this study came from regions with high burden of childhood mortality in eight countries in sub-Saharan Africa and South Asia; our sample may not represent other, lower-mortality areas in CHAMPS countries or these regions. Finally, although we included deaths from several sites, our sample size of 733 deaths with prior hospitalizations within 180 days limits generalizability of our findings.”

I appreciate the substantive revisions undertaken by the authors to improve the clarity, transparency, and reporting quality of the manuscript. However, significant conceptual and methodological limitations remain. A revision is therefore required to realign the manuscript.

Author response: We appreciate the Reviewer's comments and thought put into our revisions. We believe that we have greatly strengthened our manuscript due to the careful inclusion of their thoughtful input.
